# Tools For Building Artificial Biological Nanostructures

**DOI:** 10.1021/acsnano.5c14315

**Published:** 2026-05-28

**Authors:** Thomas S. Bradford, Sarah Hutchings, Jonathon D. Liston, Zuzanna Pakosz-Stepien, Artemis Sanderson, Ahmed Shaukat, Adam Bentham, Ting-Yu Lin, Piotr Stepien, Jonathan G. Heddle

**Affiliations:** Centre for Programmable Biological Matter, Department of Biosciences, 3057 Durham University, South Road, Durham DH1 3LE, U.K.

**Keywords:** DNA nanotechnology, DNA origami, RNA origami, protein design, engineering biology, biological nanomachines, machine learning, programmable molecules

## Abstract

Biological nanostructures and nanomachines encompass a wide range of natural assemblies from the smallest prokaryotes to viruses, enzymes, and subcellular compartments. Their capabilities are impressive, including replication, locomotion, and catalysis. To be able to design and produce modified or wholly artificial versions of such systems using biological molecules (proteins, nucleic acids, and lipids) is a long-term goal of engineering biology. However, their complexity makes the design and prediction of their properties challenging, while production, purification, and testing can also be difficult. In recent years, new approaches have been developed to facilitate these processes. Here, we review tools for designing biological molecules, highlighting their capabilities and giving examples of their successful application. Finally, we present possible capabilities of future tools and challenges to their development.

## Graphical abstract

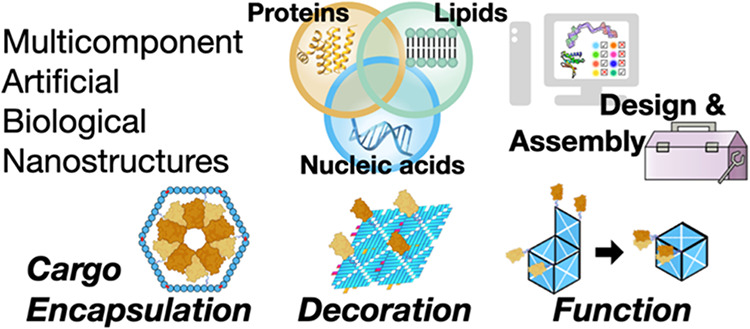

## 1 Introduction

The idea of a nanomachine seems simultaneously seductive and unachievable. It recalls the science fiction trope of a metallic, nanoscale, computer-controlled machine resembling a small-scale version of a macroscale robot. Despite considerable challenges, significant work has been carried out to try to transition this concept from fiction to reality, with notable early progress by Drexler in the 1980s.^1^ Drexler’s work included an examination of how such mechanical nanomachines could be built from precisely placed individual atoms, with the resulting machines typically bearing little relationship to biological molecules; being able, for example, to function in vacuum or even air. This approach has not been without controversy, perhaps most notably from Smalley.^2^ Regardless of the scientific and technological feasibility of such machines, what is clear is that in the decades since the idea of such “hard nanotechnology” was posited there has been little success in producing working nanomachines of the types originally envisaged.

In contrast, *biological* nanostructures and nanomachines tell a different story: In biology the concept of self-replicating sub micrometer machines able to carry out a variety of roles including synthesizing new materials, harnessing of energy from light, highly efficient catalysis of a wide range of reactions and even locomotion is very common. These biological machines are different from their hard nanotechnology counterparts: they function in aqueous solution and have structures that do not directly resemble macroscale machine components. Biological nanostructures and nanomachines have many desirable properties—they typically require low amounts of energy to function, they can self-assemble and, at the larger end of the nanoscale regime (i.e., small bacteria) can self-repair and self-replicate. Protein nanomachines are capable of performing a wide array of vital processes ranging from catalysis to transport and cellular scaffolding. Nucleic acids are also crucial: RNA has both catalytic and information encoding activity^3^ and enacts other vital functions such as relaying information between cellular compartments, while DNA acts as an information storage molecule. Finally, lipids play an important role in compartmentalization. These four materials are by their nature biocompatible and able to interface with living systems, hence they have particular promise in medical applications.

The impressive variety of useful nanostructures and nanomachines found in nature by no means represents an exhaustive library of all possible such structures. Taking only proteins as an example, an “average” 100 amino acid protein employing a library of 20 different naturally occurring amino acids would have 20^100^ possible sequences. While actual protein space may be considerably smaller than this number suggests^4^ it is quite possible, if not likely, that vast numbers of biological molecules with useful functions remain yet undiscovered.^5^

The challenge of engineering biology is to design and build functional nanostructures from biological molecules.^6^ This is a complex task, and tools are required to turn design visions into reality. Broadly, these are divided into computational, chemical and biotechnological. Computational tools utilize our accumulated knowledge of biomolecules, e.g., sequence information, bond restraints, structural contexts, to predict what structures will result from a given design or vice versa. Where these computational tools are artificial intelligence (AI) based, they extract information from large data sets, complementing physics-based methods which are based on accumulated knowledge.^7^ Chemical tools can be useful for attaching different biological materials together,^8^ such as nucleic acid and proteins, and imparting to them the ability to react to desired environmental triggers. Finally, practical biotechnological tools and approaches can address questions such as biocompatibility and immunogenicity which are important for *in vivo* applicability.^9^

Functional biological nanostructures with the potential for greatest sophistication are those which combine multiple materials (e.g., proteins, nucleic acids). The authors have been researching this new approach toward multimaterial, artificial biological nanomachines in their recently established center, giving them fresh insight into the development, advantages and disadvantages of the various tools available.

In what follows we give an overview of categories of tools that are used in nucleic acid and artificial protein design in hope of providing a useful reference for those interested in building multicomponent biological nanostructures of increasing complexity (Figure 1). The field is large, and we have taken a broad-brush approach to give readers a high-level overview of the relevant topics. Inevitably we cannot include all possible examples and where possible we prefer to include examples where our own practical experience has confirmed their utility in real world research settings. In summary, this review reflects our recent experiences of these tools and approaches we have found most useful in this blossoming research field.

**Figure 1:**
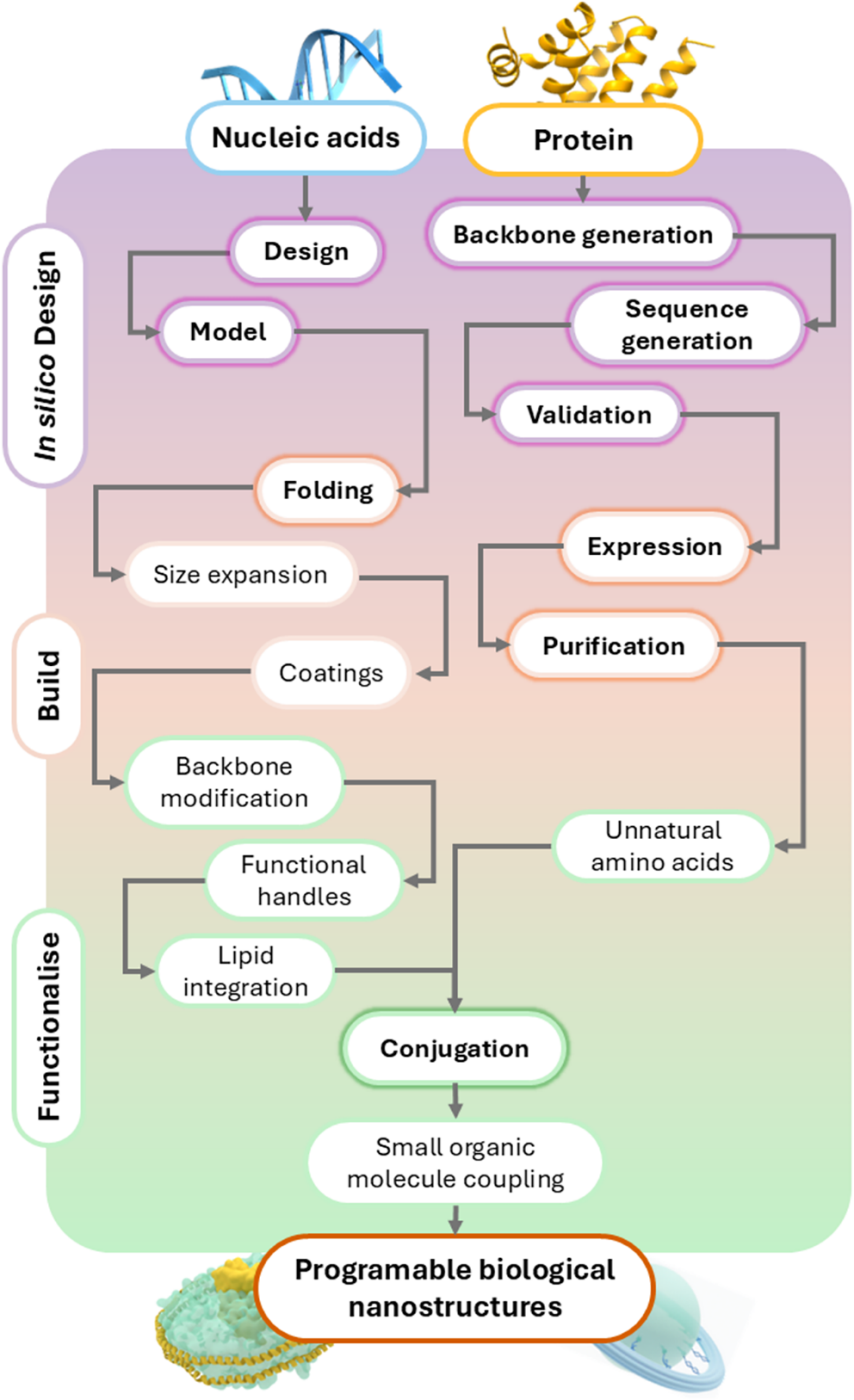
Summary of the fundamental strategies used in the design and construction of biological nanomachines covered within this review. Fundamental required stages of bionanomachine development are highlighted in bold with nonhighlighted stages being optional, dependent on the specific use case of the nanostructure being developed.

## 2 Tools for Nucleic Acid Nanotechnology

Nucleic acids are a commonly used scaffolding material for constructing artificial nanostructures. The comparatively simple rules of nucleotide-based pairing contrast with the varied, complicated interactions between amino acids in proteins. Base pairing can be employed to fold DNA into arbitrary shapes by enabling the controlled formation of DNA helices, interweaving them using Holliday junctions, and over- or underwinding the helices to introduce bends into the prepared DNA nanostructures. Similarly, RNA can also be shaped to achieve a desired geometry. Both DNA and RNA-based building strategies thus allow for bespoke creation of nanoscale constructs with nucleotide resolution. These finely manufactured assemblies can be modified with other biomolecules such as proteins, lipids, and polymers. This can tailor the function and stability of the assemblies. Additionally, programmability can be incorporated using pH-responsive DNA structures or the dynamic exchange of strands. Production is generally straightforward as nucleic acids can be readily synthesized using standard biotechnological means, as shown with mass production of DNA origami using bacteria.^10^ This section describes various methods of expanding the size of DNA assemblies, enhancing their stability, equipping them with trigger-responsive functionality and interfacing them with lipids and other molecules along with software tools allowing for facile design and prediction of nucleic acid-based nanostructures. RNA is more challenging to work with compared to DNA, but it is now also being engineered into designed structures and we introduce and describe recent developments in designed RNA assemblies and associated tools.

### 2.1 DNA Origami Design Tools: Software

DNA origami is a method of weaving DNA into 2D and 3D structures. It uses a long single strand (the scaffold strand) which is molded into the correct shape by multiple short complementary strands (staple strands). Shape control is achieved by the fact that staple strands may have complementarity to two distal parts of the scaffold strand, thus acting to “staple” them together. DNA origami structures have become highly accessible due to the development of many software tools used to both design and predict the structure of the prepared constructs. Programs allow for the assisted generation of standard and wireframe DNA origami structures (Notable examples of such programs are summarized in Table 1), these allow the routing of strands to be visualized and edited, as well as generating sequences for staples based on commonly used phage genome-based scaffolds (often built into the software) or a custom scaffold strand.

**Table 1:** A Summary of Software Commonly Used for Design of DNA/RNA Nanomachines

Software	Purpose	Features	Advantages	Disadvantages
caDNAno^11^	Designing DNA origami nanostructures	2D and 3D cylinder representation view.	Relatively easy to learn for beginners.	Can be time-consuming for large or intricate designs.
		Design with square or honeycomb lattice.	2D and 3D view, baseline software,	The user interface less modern compared to newer software e.g., no undo button
		Generates a list of staple sequences.	Square or honeycomb lattice.	Requires additional programs for atomic models and structures/PDB files.
ENSnano^16^	3D modeling of DNA and RNA nanostructures, aiming to unify different design approaches.	Adjust DNA routing in 3D view.	Can adjust DNA routing in 3D view	As a newer tool, it may have fewer established workflows and a smaller user base than some alternatives.
		Design with square or honeycomb or custom lattice.	Square or honeycomb lattice	
		Ability to color code and group sections of a design.	Can color code and group sections of a design.	
MagicDNA2 ^17^	Designing DNA origami nanostructures	Draw and adjust shapes in 3D space and apply helices to them.	Can draw and adjust shapes in 3D space and apply helices to them	The advanced capabilities can lead to highly complex designs that may be challenging to fabricate and characterize.
		Design with square or honeycomb lattice and combine both in one larger structure.	Square and honeycomb lattices	
		Features to facilitate introducing ssDNA overhangs from staples.	Automates a significant portion of the detailed design process.	
Athena^18^	Designing wireframe DNA nanostructures (double helix or 6HB sides)	View pseudoatomic and 3D structure.	Can import 3D structures	No possibility of adjusting the structure beyond the shape and wireframe type (i.e., DX or 6HB).
		Ability to import 3D shapes to convert in DNA wireframe nanostructures.	Variety of inbuilt structures	DNA overhangs need to be manually added to staples.
		Variety of inbuilt structures examples/templates.	Can export PDB file of structure.	
		Can export PDB file of structure directly.		
DNAforge^20^	Online, automated design of DNA (double strand or 6HB sides) and RNA wireframe nanostructures	Can create DNA routing and scaffold in 3D or 2D view.	Can create DNA routing and scaffold, 3D, routing and models inbuilt	Being a web-based tool, it cannot be used offline.
		oxDNA models inbuilt.	RNA nanostructure design possible	
		RNA nanostructure design possible.	Option for user-controlled GC content, vertex spacer nucleotides, staple length	
		Option for user to specify staple GC content, number of vertex spacer nucleotides, and staple length.		
		Can create DNA routing and scaffold from imported 3D mesh shape.		
		Variety of routing options inbuilt.		
vHelix^19^	Designing wireframe DNA nanostructures (double strand or 6HB sides)	Can create DNA routing and scaffold from imported 3D mesh shape.	Can create DNA routing and scaffold, 3D, routing and models inbuilt	The need for an Autodesk Maya license is a significant cost barrier.
		Can create DNA routing and scaffold in 3D or 2D view.		Requires proficiency in both Maya and the vHelix plugin.
		Variety of routing options inbuilt.		
oxDNA viewer^216^	Modeling and viewing 3D structure of DNA nanostructures	Web based program.	Web based program	Primarily a viewer and editor for a specific simulation format, not for creating designs from scratch.
		Export models generated as PDB files for figure generation.		Limited to the oxDNA representation of nucleic acids.
NUPACK^21^	Secondary structure predictions of one or mix of DNA or RNA strands	Web based program.	Can see relative Tm of various designs with mixes of individual components - useful for toeholding	Does not directly handle the design of DNA nanostructures.
		Predicts relative abundance and melting temperature of secondary structures from mixes or individual components - useful for designing toeholding systems.	Can adjust buffer Mg^2+^ and Na^+^ concentration in models	
		Can adjust buffer Mg^2+^ and Na^+^ concentration in models.		
CanDo^13^	Prediction of the 3D shape and mechanical flexibility of DNA origami structures.	Import caDNAno files to obtain rigidity predictions and generate 3D models	Provides a more accurate representation of the final 3D structure than idealized models.	Requires an input design from another program. Limited to honeycomb and square lattice design.
		Predicts the global 3D conformation of a design in solution	Allows for the evaluation of a design’s structural integrity before committing to costly synthesis.	
		Analyzes the mechanical properties and flexibility of the structure		
ROAD^100^	Designing and making RNA origami structure via cotranscriptional folding.	Designing the nanoscaffolds from a library of structure motifs, identifies potential folding issues and then performs sequence optimization tasks.	An automated pipeline and can be scaled up in size and expanded in NA modular diversity.	Pseudoknot-prediction and kinetic folding simulation are not incorporated in the design algorithm.
ssOrigami^119^	Designing and synthesis of user-specified 3D shapes from single-stranded DNA or RNA	DNA or RNA origami forming from a single stranded nucleic acid polymer.	An automated pipeline for self-folding origami.	Potential strong secondary structure presenting in the DNA or RNA strand that could cause self-complementarity.
		Can use DNA/RNA staple strands in ssRNA origami design.	Large size origami (up to 10,000 nucleotides for DNA and 6000 nucleotides for RNA).	
			In vitro synthesis.	
pyDAEDALUS^121^	Generates scaffold and staple routing for polyhedral mesh surfaces.	Designing DX-edge-based wireframe structures.	Implementing A-form dual-duplex wireframe design rules.	Not a web-based tool.
			Open-source software.	

The most popular software for generating DNA origami structures is cadnano, the original DNA origami design program, which remains a benchmark for newer tools.^11,12^ Cadnano allows helix-by-helix design of structures on one of two fixed latices (honeycomb or square) (Figure 2A & B) and aids the user with staple routing and generation of staple sequences, however, the structure’s overall design still needs to be conceived and mapped out manually, including scaffold routing and any DNA overhangs to be used for hybridization-based attachment of other molecules. Cadnano is useful for designing 2D origami structures and modifying existing designs but with some experience can be used to prepare complex 3D structures. It is able to output designs as a 3D model wherein cylinders represent each DNA helix. These can be viewed using CanDo which also predicts the rigidity of designed structures.^13^ Cadnano files can also be used in oxDNA^14^ an online molecular simulation tool wherein a 3D model of the structure can be created, which can be adjusted by users via extending, cleaving or joining DNA sequences and modeling simulations can be run. The oxDNA files can be converted to PDB (Protein Data Bank) files using an online tool (tacoxDNA^15^) and be used for visualization with standard molecular visualization software. Both CanDo and oxDNA are convenient tools for the general validation of designs and the creation of high-quality figures. The key difference between the two is that CanDo utilizes a finite element modeling (FEM) framework to treat DNA as elastic beams, facilitating rapid prediction of equilibrium geometries, mechanical strain, and root-mean-square fluctuations (RMSF). In contrast, oxDNA is a coarse-grained molecular dynamics (MD) simulator that models DNA at the nucleotide level, capturing stochastic thermal fluctuations and thermodynamic transitions like base-pair melting. While CanDo typically outputs PDB files and heatmaps of structural flexibility, oxDNA generates comprehensive trajectory files (configuration and topology) that can be converted into PDB or LAMMPS formats for advanced visualization and ensemble analysis.

**Figure 2:**
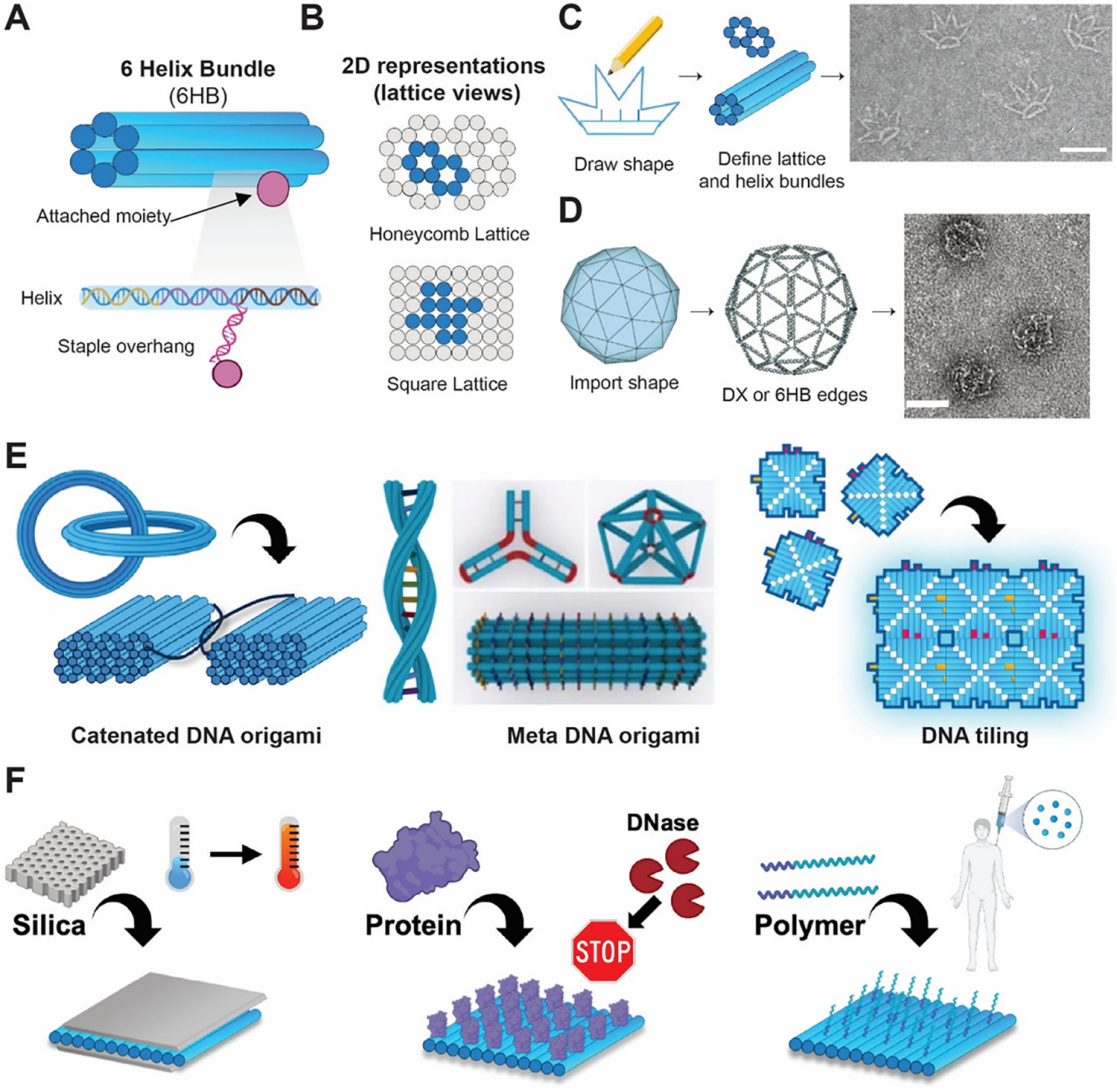
DNA origami: construction, enlargement and coating. (**A**) DNA origami is made of bundles of DNA helices that are typically arranged in a honeycomb or square lattices; “6 helix bundles” (6HB; shown as cylinders) are a common honeycomb-lattice motif. By extending selected staple strands at desired sites, molecules bearing complementary DNA handles can be attached via base pairing, adding functionality to the nanomachine (e.g., protein, lipid, or small molecules; shown as a red circle). (**B**) DNA nanostructure design software typically contains 2D and 3D representations of the design. Some modeling tools allows viewing or modeling with nucleotide resolution. 2D views can be taken looking at the end of the helixes to show the lattice design. This is often based on a honeycomb or square lattice, but freeform lattices have also been used. (**C, D**) Typical DNA nanostructure design pipeline involves designing a shape, deciding on the method to construct this as origami or wireframe origami and then designing it *in silico*. Some DNA nanostructure design software allows the user to draw a shape, assign the lattice, choose a scaffold sequence and will then create staple strands from this (**C**). Transmission electron microscopy (TEM) image reprinted from ref 17. (Copyright 2023 The American Association for the Advancement of Science). This shows a structure designed in this way using MagicDNA2. (**D**) Wireframe design software, and some origami software, allows users to import a 3D shape model (or use an existing template), choose whether the edges are made of two helices or 6HB, and will then create a scaffold routing and staple strands and choose a sequence for the scaffold. Example of structures designed in vHelix and imaged with TEM reprinted from ref 19. (Copyright 2015 Springer Nature) are shown. (**E**) Strategies to expand DNA origami size beyond scaffold length. Catenated scaffolds (left) use covalently linked DNA to increase size. Meta-DNA structures (center) employ DNA origami bundles as building blocks, reprinted from ref 35. (Copyright 2020 Springer Nature). DNA tiling (right) creates diverse 2D arrays, yielding larger structures. (**F**) Strategies for applying coatings to DNA origami. Examples of coatings with a porous silica material, a protein, and a polymer, highlighting the versatility of modifying these nanostructures.

For larger or more complex structures, more advanced 3D structural visualization may be helpful. ESnano^16^ builds upon the capabilities of cadnano. It allows the use of any 2D cross-sectional lattice rather than being restricted to honeycomb or square, facilitating preparation of more complex designs. ESnano can also adjust the 2D strand routing in a 3D representation and allows for more detailed 3D visualizations as instead of cylinders, helices are viewed with nucleotide resolution. This makes inspecting and adjusting the cross over points between helices easier as the turn of the DNA helix can be seen. This program streamlines the design process with features for color coding and grouping of sections of a design, which may be useful in larger structures.

MagicDNA2 ^17^ is based on cadnano with oxDNA modeling built in. It allows the user to draw and adjust shapes in 3D space as a series of straight or curved lines and to subsequently apply to them a selected number of helices arranged in square or honeycomb lattices (Figure 2C). It also allows the user to easily add features such as ssDNA overhangs—useful for joining multiple DNA structures to form larger structures from multiple origamis.

Wireframe design software has similar capabilities to origami software. An example is ATHENA^18^ which combines multiple text-based software for converting meshes to 2D and 3D. ATHENA has a basic GUI and allows designs on either a duplex or six helix bundles for each wireframe edge. Like cadnano, helices are represented in 3D as cylinders, but the strand routing can be overlaid on the 3D representation. A pseudoatomic representation is also inbuilt, and a PDB file can be exported if desired. Staple sequences can be generated based on a default or a custom scaffold uploaded to the software. For simple wireframe designs, ATHENA may be appropriate, however the DNA overhangs have to be designed and added manually into the staple DNA sequence. CanDo and oxDNA can be used for modeling and predicting structural flexibility of designs.

For more complex wireframe designs or to have more options when building a structure including inbuilt oxDNA modeling, software such as DNAforge, vHelix and others exist.^19,20^ Both DNAforge and vHelix (Figure 2D) allow the user to create DNA routing and scaffold and staple sequences to make a DNA wireframe structure from a 3D mesh shape. DNAforge allows 3D visualization, a routing mode, cylinder viewing and nucleotide and atomic models. Multiple design and routing methods are available for building the DNA sequences onto the wireframe mesh. Alternative designs and routing methods available in these programs may be more suitable for nanostructures that need to form or function in certain conditions—for example lower divalent cation concentrations in physiological conditions. DNAforge also allows building of RNA scaffold designs. Maximum and minimum staple length, spacer nucleotides vertices, and GC content (in some building models) can be adjusted to the user’s specifications.

When designing very small structures, or planning sequences for hybridization, toe-holding or strand blocking (see Section 2.5), NUPACK^21^ can be useful. This online tool provides secondary structure predictions of DNA and RNA strands alone or in mixtures at various salt concentrations and a temperature range chosen by the user. This prediction of how a mixture of strands will interact allows a designer to change the sequence of each strand to adjust the relative melting temperature of complexes to finetune their interactions before the final design is tested *in vitro*.

At the forefront of the currently developing DNA nanostructure software are new AI-based approaches to origami design. However, these are limited by the relative lack of structures; needed for use as training data sets. To solve this problem, most systems rely on a combination of both new AI-techniques with previously established computational modeling. The ability to predict a DNA origami structure from staple and scaffold sequences has been tackled using non-AI computational approaches (e.g., REVNANO^22^). More recently, DeepSNUPI is a model that combines graph neural networks with physics-based approaches to achieve inverse design of DNA origami—enabling rational design of complex nanostructures.^23^ Mango is a recent example in which wireframe DNA origami structures can be produced using a generative process, thereby avoiding the requirement for manual design of the target shapes.^24^

Future software may expand upon the ability to draw or upload shapes from which designs can be automatically generated while adding or integrating tools for strand extension to combine discrete DNA building blocks or sections of other complex structures. Programs which increase the ease with which users can create large origami structures out of multiple origamis may also be a welcome addition. As new innovations in DNA nanostructure design evolve, their inclusion will help bring the tools to an even wider audience as DNA nanotechnology becomes a more mainstream approach for investigating a variety of scientific questions.

### 2.2 DNA Origami Design Tools: Expanding Size

Standard DNA origami structures are made from a scaffold strand approximately 7 kilobases in size. Larger DNA structures may be desirable for some applications and two primary strategies can be employed to achieve this: using longer DNA scaffolds or using hierarchical assembly of DNA origami monomers. Using longer DNA scaffolds can be a straightforward approach with synthesis possible through techniques such as PCR, rolling circle amplification, or extraction from bacteriophages.^25^ However, there are notable limitations: As the scaffold length increases, the formation of disruptive internal secondary structures becomes more likely, hindering proper folding and reducing efficiency. Additionally, longer scaffolds are more expensive to produce. An alternative approach involves using catenated DNA strands (Figure 2E, left), where single-stranded DNA is covalently linked, allowing each strand to independently fold into DNA origami and attach to others, potentially improving overall stability, so-called “DNA-Topogami”.^26^

Hierarchical assembly offers a more cost-effective solution for constructing large DNA structures. This method involves the concept of modularity, meaning assembling smaller DNA origami monomers into larger superstructures.^27^ Hybridization and base stacking are the primary driving forces. Hybridization utilizes DNA origami units with complementary “sticky ends” that assemble based on sequence-specific Watson–Crick base pairing. This approach has been used to form numerous structures, including polyhedra, 1D ribbons, and 2D lattices.^28^ While longer scaffolds achieve high yields for structures up to 100–150 nm (∼12,000 nt), efficiency drops significantly as they approach 500 nm due to misfolding and kinetic traps.^29,30^ Conversely, hierarchical assembly enables scaling to micrometer dimensions (>100,000 nt), but typically results in lower cumulative yields due to the complexities of multistage purification and secondary assembly.^31^ Base stacking takes advantage of π–π interactions between terminal base pairs at blunt-ended helices,^36^ creating short-range, largely sequence-independent contacts that align and stabilize DNA origami–origami shape-complementary interfaces and promote higher-order assembly, with notable examples including 3D origami crystals,^32^ dynamic 3D multilayered structures^37^ and arrays.^34^ Hybridization and base stacking can be used together to construct intricate 3D assemblies.^33^

Additionally, it is possible to increase the scale of these constructions to create much larger DNA nanostructures formed of individual components, created themselves via the previously described DNA sculpting techniques. These are then joined together using methods such as DNA tiling, whereby traditional DNA origami tiles are stitched together to form new structures of significantly increased size,^34^ and meta-DNA origami^35^ (Figure 2E, center and right) where double helices are formed out of simple origami based backbones exhibiting pseudobase pairing interactions using specifically designed staple strand overlaps. This allows them to mimic single-stranded DNA behavior, forming much larger double helices with similar programmable properties to their smaller counterparts but on a much larger size scale. Both these techniques represent commonly utilized mechanisms to increase the previous size limitation of DNA origami. However, there are of course many other strategies that go beyond the scope of this review.

### 2.3 DNA Origami Function: Interaction with Membranes/Lipids

Lipids are essential biological building blocks that form membranes defining cellular and subcellular boundaries. A comprehensive overview of lipid nanostructures is beyond the scope of this review, and here we focus on actionable, robust ways to incorporate lipids into bespoke artificial nanostructures. Protein–membrane interactions are powerful, enabling straightforward membrane localization via genetically fused amphipathic membrane-targeting helices,^38^ biotin–streptavidin-mediated attachment to biotinylated lipids/liposomes^39^ or engineered single-pass transmembrane anchors. These approaches, however, primarily provide attachment to pre-existing membranes,^40^ whereas programmable 3D membrane seeding and shape control required by advanced designer nanosystems is currently more readily achieved with DNA-based approaches. While AI-designed proteins may close this gap, DNA nanotechnology remains a leading route for precise membrane interactions and shaping in artificial nanostructures. Integration of DNA origami with lipids can be achieved via electrostatic interactions between lipids and the negatively charged backbone of DNA (Figure 3A), enabling DNA origami to interact with both supported planar lipid membranes and cell-sized vesicles. These interactions can be exploited to organize lipid-bound DNA origami into arrays^41^ and control its distribution between phase separated islands of lipids with varying lipid compositions. The formation of these arrays has a strong dependence both on the type of lipids used and the ion concentration.^42^ Cationic lipids can also be used; due to the complementary charge. These can coat DNA origami, potentially useful for cell penetration and increased stability.^43^

**Figure 3:**
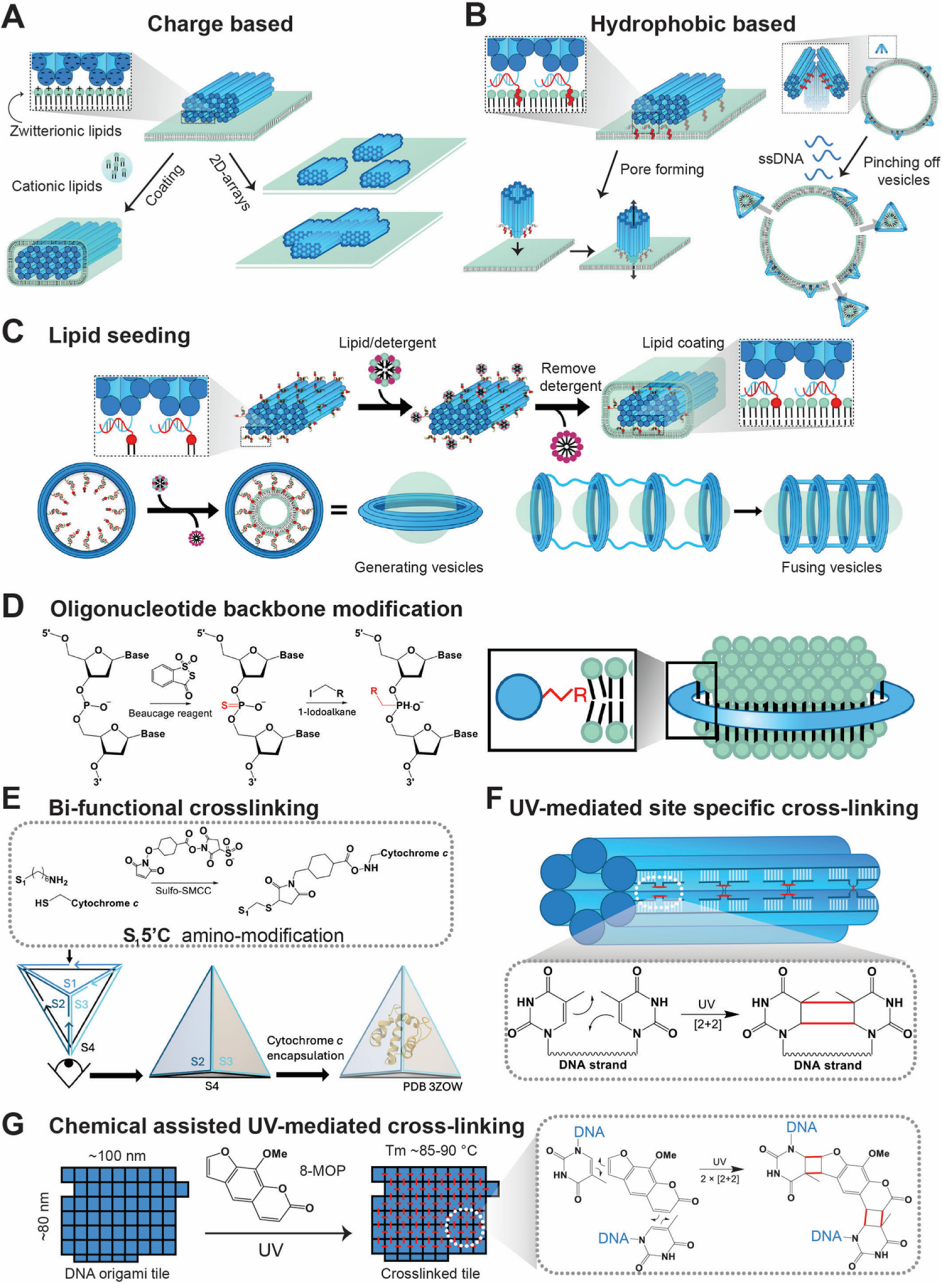
Simplified schematics showing a selection of commonly utilized DNA modification tools for both DNA-lipid interactions and chemical functionalization strategies. (A–D) A selection of methods enabling the coating of DNA nanostructures with various membranes. (A) The interaction with zwitterionic lipids to enable their placement on lipid bilayers and arranging them into 2D arrays or using cationic lipids to coat DNA nanostructures. (B) The use of hydrophobic moieties, such as cholesterol, as covalently bound anchors for lipid coating, piercing membranes or pinching off vesicles. (C) Various lipid seeding methods that can be used to coat DNA nanostructures in lipids, generate vesicles with them and manipulate vesicles by physically fusing them. The lipid conjugated with a DNA oligo is depicted in red, and complementary sequence of the oligo to the DNA origami enables the site-specific lipid seeding. (D) Mechanism of the use of oligonucleotide backbone modification with iodo-functionalized carbon chains for the creation of localized lipophilic environments - adapted from work by Iric et al.^54^ (E) Scheme illustrating the use of heterobifunctional cross-linkers for protein to DNA bioconjugation, with an illustrative example from Erben et al. (PDB 3ZOW).^55^ The oligo strands (S1–S4) are indicated while the cytochrome c protein is shown in a cartoon representation. (F) Schematic representation of site-mediated UV cross-linking of DNA nanostructures for increased stability, as reported by Gerling et al.^231^ (G) Mechanistic explanation of 8-methoxypsoren (8-MOP) assisted UV cross-linking for increasing thermal stability of DNA origami, based on data from Rajendran et al.^57^

Another important modality for interfacing DNA origami with lipid membranes is the utilization of DNA extension strands equipped with hydrophobic modifications (Figure 3B). This can include lipids, cholesterol, tocopherol, and short acyl chains, with their placement being on either the 3′ or 5′ termini as well as midchain, where they can be added by modification of SH groups introduced into the phosphate backbone. This modification can be used in distinct ways: the first is the direct attachment of DNA origami to lipid membranes, where the hydrophobic membranes can be punctured with the origami DNA constructs, generating membrane-protein-like assemblies. These can be used as artificial channels that control the transport of substances across the membranes.^44^ Hydrophobic modification can also be used to shape lipid membranes, controlling their curvature to match the curvature of DNA origami^45^ or to “pinch-out” lipid vesicles, resembling the generation of vesicles by cells.^46^

The second use of hydrophobically modified DNA origami is as seeding domains for lipid membranes with the shape dictated by the shape of the DNA origami (Figure 3C). The seeding domains are typically phospholipids covalently attached to DNA origami; they serve as a nucleation site for the formation of bilayers which is achieved by addition of lipid/detergent micelles and subsequent removal of detergent. Use of seeding domains can result in total encapsulation of the seeding DNA origami in a way reminiscent of encapsulated viruses.^47^ The seeding domains can also be used to facilitate generation of lipid vesicles within the ring-shaped DNA origami. (Figure 3C).^48^ The spacing of the generated vesicles can be controlled using assemblies of multiple DNA origami structures, which can be fused to generate tubular lipid structures reminiscent of tendrils. The final modality is the direct attachment of lipid-binding proteins, typically nanodiscs. These can be attached to DNA origami^49^ or used as different lipid seeding particles to generate planar lipid membranes encircled by a DNA origami structure.^50,248^

### 2.4 DNA Modification

Techniques such as DNA origami allow us to design and build complex DNA-based structures. However, some desirable functionalities are not possible using the DNA alone and require attachment of other molecules (e.g., proteins, fluorophores *etc*.). Fortunately, DNA modification chemistry is an advanced field providing many pathways to functionalization, some of which are reviewed below.

#### 2.4.1 Handles for Postsynthesis Functionalization of DNA Nanostructures

The development of bottom-up oligonucleotide synthesis began in the 1950s.^51^ The subsequent progress made in phosphonamidite chemistry represented a revolution in bionanotechnology—providing access to explicitly designed DNA strands with the selective reactivity required for use as building blocks of more complex nanomachinery. In brief, in-synthesis modification of DNA strands allows for the incorporation of any chemical functionality compatible with the synthesis conditions and structure of the protected oligonucleotide strand, provided it can be coupled to a phosphonamidite group, permitting incorporation of non-naturally occurring nucleotides. However, for the many functional groups that are incompatible with solid-phase synthesis or for conjugation to other biological components of the target nanomachine—such as proteins—phosphonamidites containing so-called “functional handles” may be incorporated to allow for postsynthetic functionalization and bioconjugation (Figure 3D–G). Goodchild provided an excellent review in 1990 of both in-synthesis and postsynthesis oligonucleotide modification tools. Specifically, they provide an overview of the available synthetic strategies for the creation of modified oligonucleotides, for use in conjugation of additional targets of interest to the oligonucleotide-incorporating different features within the same product. This remains an invaluable resource to this day.^52^

The addition of in-sequence functional handles during strand synthesis allows for precise control of further functionalizations and bioconjugations. However, the creation of multicomponent bionanomachines from presynthesized or assembled DNA objects often relies on postsynthesis modification being carried out in aqueous solutions with free, charged DNA. This approach imposes significant limitations on the range of applicable chemical processes that can be utilized, with solubility of the reaction partners posing a significant hurdle. A “toolbox” of the most common functional handle reactions available to the designer is shown in Table 2. Chemical attachment methods which are typically restricted to nucleic acids, such as thiol-based Michael addition and Dials Alder reactions, are presented in detail. While enzymatically catalyzed systems—such as sortase and protein biotinylation—are also widely used, their details are more extensively covered in Section 3.4 due to their ability to be incorporated from the protein side of the coupling, on account of the milder reaction conditions. Marked entries (*) within the table illustrate functional groups which can be incorporated within both the oligonucleotide sequence as the functional handle, or can be within a partner molecule (protein, fluorophore, etc.). For more comprehensive information on the relevant reaction chemistries, see Madsen and Gothelf in their excellent 2019 review.^53^

**Table 2:**
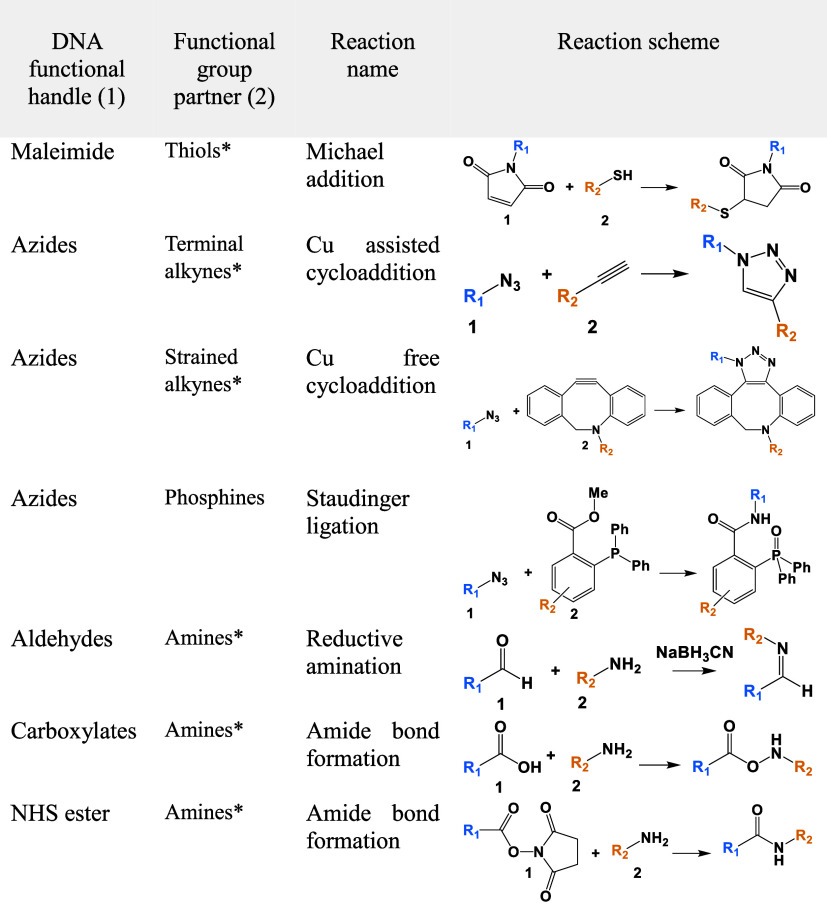
A Selection of the Most Utilized Chemical Reactions Available for Post-Synthesis Oligonucleotide Modification and Biomolecule (R1 and R2) Conjugation^a^

a Functional group partners marked with (*) can also be incorporated into the oligonucleotide sequence as the functional handle for the reaction, whereas non-marked moieties can only be present in the reaction partner (protein, fluorophore, etc.). Additional reactions, more closely aligned with protein labeling, may also be utilized to combine DNA with other biomolecules—these are covered in further detail in Section 3.4.

#### 2.4.2 Backbone Modification

In addition to the in-synthesis incorporation of functional handles within the oligonucleotide, modifications to the phosphate backbone of the structure can also be exploited. The most relevant of these for bionanomachine functionalization is the in-synthesis formation of phosphorothioates. In this process the standard oxidation stage of strand synthesis is replaced by a sulfonation step with Beaucage’s reagent (Figure 3D). The incorporation of phosphorothioates allows for alkylation of DNA nanostructures at targeted points along the helix, resulting in easily controllable site-specific functionalization. This technology is exemplified in the recent creation of DNA-encircled lipid bilayers (DEBs);^54^ a DNA nanotechnology used in the biophysical investigation of membrane proteins by Iric et al. Therein, DEBs are created by first reacting short chain alkyl-iodides with oligonucleotides containing a small number of phosphorothioates that are subsequently incorporated into a circular single stranded template, creating a double stranded DNA minicircle with small carbon chain decorations along the inner circumference (Figure 3D). This technology enables capture of phospholipid and the development of an encased membrane with the ability to stabilize membrane proteins for further study.

#### 2.4.3 Covalent Attachment Methods

DNA nanostructures modified with appropriate “handles” can subsequently be used for covalent attachment to other molecules of interest. One of the simplest, nonsite-specific ways to covalently attach DNA nanostructures to proteins is via the use of heterobifunctional cross-linkers; most commonly consisting of both maleimide and *N*-Hydroxysuccinimide (NHS) ester derived functionalities. Hereby, each side of the molecule will specifically react with either a specifically designed thiol/amine functional handle incorporated within the DNA nanostructure, or available surface lysine/cysteine residues within the target protein—forming a covalent link between them. These nonspecific methods have the benefit of not requiring extensive protein engineering steps, due to their reliance on the sulfur/amine chemistries of the naturally occurring surface residues of the target protein. This technology was used to great effect in the encapsulation of cytochrome *c* within a tetrahedral DNA cage, using sulfo-succinimidyl-4-(N-maleimidomethyl)­cyclohexane-1-carboxylate (sSMCC) as the cross-linker^55^ (Figure 3E). Here, Erben et al. create a DNA tetrahedron via the self-assembly of four oligonucleotide strands, where strand 1 (S1) is functionalized with an amino functional handle at the 5′ end—facing into the tetrahedron. Using sSMCC, S1 can be covalently attached to exposed surface cystine residues on cytochrome *c* before assembling the tetrahedron by annealing with strands 2–4 (S2–S4). This results in encapsulation of the protein within the DNA cage, posing exciting possibilities for DNA nanotechnology in drug delivery and cargo transport systems.

In addition to bifunctional cross-linkers for nonspecific attachment of functionalized oligonucleotides to other biomolecules, other tools are available for more site-specific attachments, such as the use of azide–alkyne cycloadditions. The approach utilizes terminal or strained alkyne functional handles within the DNA that react with azides added to the protein sequence, connecting the two biomolecules via a triazole linkage. A summary of the chemical considerations involved in these types of reactions can be found in a review by Pickens et al.^56^

One of the main shortcomings of cross-linking DNA to other biomolecules using sulfur-chemistry based methods is the lack of specificity. This often necessitates extensive purification to remove multiple conjugates with excessive cross-linker. To avoid this, DNA can be added to specific locations within the protein of interest by utilizing enzyme catalyzed coupling reactions, such as microbial transglutaminase and sortase-mediated reactions. These methods, alongside additional noncovalent methods such as the exploitation of the biotin–streptavidin interaction will be covered in greater detail in the subsequent protein modification tools section (Section 3.4) of this review.

#### 2.4.4 Stability Modulation

Stabilizing DNA origami structures is essential for their application in nonideal environments such as *in vivo*. Several approaches have been developed to improve the structural integrity and durability of DNA origami, broadly categorized into chemical cross-linking and noncovalent coatings (Figure 2F). These methods aim to enhance resistance to heat, mechanical stress, low cation concentrations, and biochemical degradation, such as exposure to nucleases.

Chemical cross-linking is a common strategy that involves forming covalent bonds between DNA strands. This typically increases the thermal stability and resilience of the DNA structures. Cross-linking agents, activated by UV light or specific chemical reactions, create additional bonds between pyrimidine bases or other functional groups in the DNA, reinforcing the structure (Figure 3F). This type of stabilization can be achieved through various chemical agents, such as 8-methoxypsoralen (8-MOP)^57^ or 3-cyanovinylcarbazole,^58^ which significantly improve the structure’s resistance to denaturing conditions such as heat and chemical stress (Figure 3G), with increases of thermal degradation resistance of up to 30 °C seen for tile-based structures, as well as dramatic increases in mechanical strength as measured by the Derjaguin-Muller-Toporov (DMT) modulus doubling from 100 to 200 mPa.^59^ Moreover, hybrid methods that combine cross-linking with other stabilization techniques have been shown to increase durability, particularly in hostile environments like low magnesium concentrations or cell culture media.^60^ Although chemical cross-linking shows promise for stabilization of DNA nanostructures, it may impact performance. For instance, a cage made of DNA origami, designed to open when specific conditions are met, may have its function limited if the covalent bonds formed by the chemical cross-linkers prevent the dehybridization of key locking strands. In such instances noncovalent coatings may be preferable.

Alternative approaches for improving stability include biomineralization,^61^ electrostatic interactions,^62^ and protein-based coatings.^63,64^ For example, biomineralization with calcium phosphate not only enhances thermal stability, but also strengthens the mechanical properties of DNA structures.^61^ Electrostatic coatings leverage the negatively charged DNA backbone to bind positively charged molecules, providing stabilization against environmental stressors like heat, low cation levels, and enzymatic degradation. This method is particularly useful for biological applications, as it improves the structures’ survival in cell media and increases resistance to nucleases.^62^ Protein-based and polymer coatings offer additional advantages, particularly in biological contexts (Figure 2F). Coating DNA with dendron-protein conjugates^63^ or lipid bilayers^64^ has been shown to enhance stability, biocompatibility, and resistance to nucleases, while maintaining functionality for biomedical applications such as drug delivery or gene editing. In particular, encapsulation in lipid bilayers^47^ or dendritic oligonucleotides^65^ allows for both structural integrity and potential interaction with biological systems, improving circulation times and transfection efficiency in cellular environments. In nonbiological applications, materials like silica, aluminum oxide, and graphene have been used to coat DNA origami, enhancing both thermal and mechanical stability.^66^ These coatings allow DNA structures to withstand extreme environmental conditions such as high temperatures, mechanical stress, and long-term storage.^67^ Silica coatings, in particular, provide long-lasting stability in aqueous solutions and improve mechanical properties, such as rigidity and compression resistance.^68^ Additionally, the use of silica as a coating material has been shown to preserve the addressability of designed single stranded protrusion sites, maintaining the functionalization capability of the nanostructure while still incurring the previously discussed stability benefits of surface coating.^69^

### 2.5 Programming DNA Origami

Biological molecules often require conformational changes to carry out their function and there is a desire to replicate these features in artificial DNA nanostructures. Programmable elements can range from simple fluorescence cycling caused by alterations in local environment, to triggerable docking of specifically designed nanostructures to surfaces. This has been extended to more dynamic, multifunction, DNA nanostructures such as hollow DNA cages (used for the encapsulation and cell-specific delivery of drug-like molecules to various cells), molecular walkers (capable of independent movement across a surface), and molecular motors.^70,71^

One example utilizes the manipulation of the intermolecular distances between specific sites within a DNA nanostructure to alter the level of Förster resonance energy transfer (FRET) observed between two attached fluorophores.^72^ This allows the nanodevice to be used as a molecular sensor—whereby association with an external molecule causes an alteration in the DNA structure, changing the distance of the FRET pair, resulting in a shift in measured signal intensity. The high degree of control and specificity during the design process of DNA nanostructures has recently provided incredibly precise levels of control over this style of biosensor technology; allowing for modulation of both onset and sharpness of the sensor response window as well as sensor specificity.^73^

Conformational changes in DNA nanostructures can be activated using different triggers, which are briefly explained below. One method is to incorporate sites for specific enzymatic cleavage (by restriction enzymes) or extension (e.g., by telomerase) of DNA strands (Figure 4A). This approach can be used to increase the length of strands or to allow parts of a structure to move apart from each other—a feature useful for opening a cage or dynamic control of a structure’s shape.^74^

DNA base pairing can be taken advantage of to bestow DNA nanostructures with programmable features so that they respond to an external trigger in a predefined way. Two DNA strands containing mismatches can still base pair in some conditions, such as in the presence of other, supporting strands or at a lower temperature. These strands can disassociate from each other by competition with a more energetically favorable DNA complex—for example by replacing the mismatched DNA sequence with a fully complementary one (Figure 4B). These mechanisms can be used for on-demand “unlocking” to release DNA or RNA cargo from a structure, or to release a connection between parts of an origami design to trigger conformational change. “Toe-holding” is one of the key techniques used for strand displacement whereby a majority of an oligonucleotide sequence is base-paired to a complementary sequence, but a section remains unpaired, the so-called toe-hold sequence (Figure 4B). The addition of another strand complementary to the full sequence can bind to the toe-hold, replacing the original paired strand. This allows strand replacement without need for mismatched sequences, with the reaction progress driven either by a net increase in base-pairing (enthalpy-driven) or the dissociation of a multicomponent complex into a greater number of individual molecules (entropy-driven). The kinetics of this process are highly tunable based on toehold length, sequence composition, and the operating temperature relative to the toehold’s melting temperature. The online tool NUPACK may be useful for comparing the relative concentration of different DNA complexes in mixtures of strands at a range of temperatures, allowing facile toe-hold design preparation and evaluation. In the years since toeholds were first developed by Yurke et al. to create DNA tweezers, this process has been used to achieve effects such as opening of a DNA cage or amplification of a signal upon addition or sensing of a DNA strand.^75,76^ One fascinating example of the toehold approach was as a means to control opening of the gate of a protein-transporting membrane channel constructed from DNA,^77^ where a DNA “trapdoor” covering the channel could be reversibly opened by addition of toehold strands, with the opened version allowing transport of cargoes such as trypsin and GFP across the membrane. Additionally, one of the most promising applications of this technology developed over the last two decades, DNA walkers, utilize toehold mediated strand displacement as their main driving force for use in both nanoscale signaling and cargo transport.^78^

**Figure 4:**
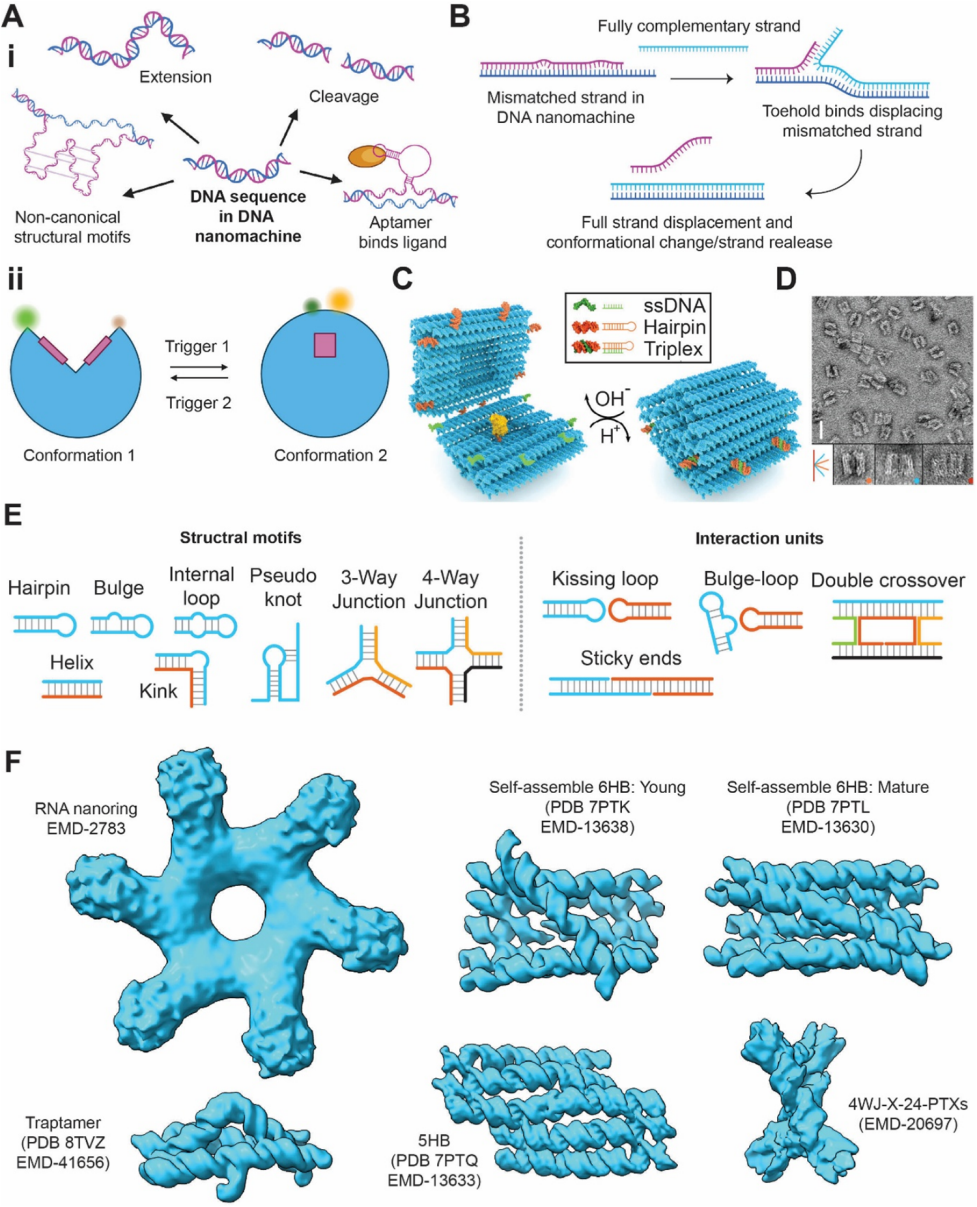
Elements for DNA nanostructure programmability and examples of RNA nanostructures. (**A**) (i) Examples of how programmability can be incorporated into DNA nanostructures at the sequence level. By extending or cleaving strands, distances between parts of the nanomachine can be changed, allowing movement. Formation of aptamers upon ligand binding or formation of noncanonical structures (e.g., i-motifs and triplexes) based on ion concentration or pH can also be used to disrupt base pairing, changing how the DNA structure folds and the overall shape of a nanomachine. (ii) By adding a trigger (e.g., pH, ion concentration, new DNA strands) conformational change of a DNA nanostructure (blue circle) can be achieved by formation of disruption of a structure as shown. This can be used to change output of a FRET pair attached to the nanomachine by changing their intermolecular distance. (**B**) Toeholding is a technique used in some DNA nanomachines to trigger conformational change or turn on or off a signal. A DNA strand is bound to another strand which is not fully complementary, and leaves a single stranded region unbound. When a strand which is complementary to the whole sequence is added, it can first bind to the exposed single stranded region (toehold) and then displace the imperfect complement strand which is then released. (**C**) An example structure of a DNA nanomachine from Ijäs et al.^70^ A cargo is bound via an attached DNA strand binding to its complement. Upon pH change ssDNA on one-half of the cage forms a triplex with a hairpin on the other half of the cage. This closes the cage, protecting the cargo. (**D**) TEM images show the cage in open, half open and closed conformations. (**E**) Cartoon depiction of DNA and RNA as double-stranded and single-stranded, respectively. 2D structures of RNA structural motifs and interaction units for RNA nanostructures (right). (**F**) Examples of experimentally determined 3D maps of artificial RNA nanostructures. HB: helix bundle; PTXs: paclitaxels. Panels C and D reprinted from Ijäs et al.^70^ (Copyright 2019 American Chemical Society).

More unusual DNA conformations can be used in conjunction with the toe-holding and strand replacement techniques. Aptamers are DNA or RNA sequences that are created using an *in vitro* selection method known as systematic evolution of ligands by exponential enrichment (SELEX).^79^ This results in DNA or RNA molecules having 3D structures that can bind to a specific ligand. A number of other methods for creating aptamers are detailed in other reviews.^80^ As aptamers are nucleic acids, they can be readily incorporated in origami constructs using simple hybridization. They can provide origami with ability to bind to selected targets, but can also designed with more complex functionality. One example is addition of a ligand used to outcompete a interaction with a second DNA strand to trigger a conformational change, such as opening of cages,^81^ e.g., for targeted delivery of cargo to certain cell types.^82^ Cascades of aptamer binding, releasing toeholds, has allowed the creation of basic logic pathways to combine signals and reduce off target binding to cells.^83^

Programmable elements can also be added to nanostructures with use of DNA triplexes employing Hoogsteen base pairing, I-motifs and other noncanonical DNA structural motifs (as discussed in depth in other reviews^84^). These motifs can only form at specific pHs or in the presence of certain ions, and these conditions can, therefore, be used as triggers (sometimes reversible) to control these motifs and induce larger conformational changes (Figure 4C,D). Formation of the structural motifs can bring together distal parts of a nanostructure as exemplified with a pH change to trigger cage opening and closing or a fluorescent signal change as FRET pair alters intermolecular distance.^70^

Creation of complex artificial DNA nanomachines has been realized; for example, a rotary apparatus has been demonstrated.^85^ By combining their programmability with other functional elements, such as attached proteins, the potential is vast and may be useful for a variety of medical and industrial applications in the future.

### 2.6 RNA Nanotechnology

There are analogies between RNA and DNA nanotechnologies in that they can both be programmed into desired shapes using staple strands via canonical Watson–Crick base pairing. RNA can be chemically modified to achieve desired assembly or function via click chemistry to conjugate chemical groups (e.g., fluorophores^86^) or to incorporate chemically modified nucleotides^87^ (e.g., biotinylated NTPs or aminoallyl-NTPs) during synthesis.^88^ This has been demonstrated in work generating a synthetic functional hammerhead ribozyme through linking RNA strands via their backbone instead of phosphodiester groups. This synthetic and unnatural triazole linkage locates in the catalytic pocket opposite to the cleavage site, mimicking the catalytic fold of the ribozyme.^88^ This exemplifies the potential for designing functional synthetic RNAs based on biological examples in cells. As RNA is inherently less stable than DNA, enhancing stability is a key factor commonly achieved via chemical modification of the nucleotides. For instance, 2′-fluoro (2′-F) modifications of RNA nanoparticles:^89^ 2′-F can support RNA duplex A-form configuration via the C3′ endo conformation of the sugar ring,^90^ helping resist both nuclease and base degradation. RNA nanotechnology also has been developed for medical applications where unwanted immune responses are a general concern. RNA modifications,^91^ such as pseudouridines or 2′-*O*-methyl (2′-OMe) have both been shown to assist with this problem. Hence, chemical RNA modification have been introduced to RNA nanostructures to expand their potential applications^92^ (see review^93^).

RNA/RNA or RNA/DNA hybridization-mediated structure formation is more complex than seen for the DNA/DNA double helix: When RNA hybridizes with DNA or RNA, it forms an A-form helix due to its 2′-hydroxyl group on the sugar that causes C3′-endo formation. RNA can self-assemble into secondary structures, such as hairpins, stems, and loops, and further 3D conformations (Figure 4E).

Many naturally existing “RNA brick” structures have been discovered and archived in databases along with the experimentally determined information on RNA–RNA and RNA-protein interactions at the molecular level (Figure 4E).^94^ Crucially, RNA can display catalytic activities, as demonstrated by ribozymes, riboswitches, and Y RNA. These naturally existing RNA structures can be used in RNA nanotechnology as building blocks^95^ exploiting both canonical and noncanonical base pairing and base stacking interactions (Figure 4F).^96^ Catalytic RNA 3D motifs, such as RNA aptamers, have been incorporated into engineered RNA nanostructures to increase functional capacity.^97,98^ Moreover, long natural RNAs (e.g., mRNA or rRNA^99^), produced in cells via cotranslational folding have been used as scaffold RNA materials for RNA nanotechnology.^100–102^ Many assembled bespoke RNA 3D structures^103^ show increased structural plasticity and stability, contributing to nanomedicine applications and development, such as drug delivery, which has been demonstrated using mammalian cells.^97,104–106^

Many designed or experimentally obtained structures are deposited in publicly available databases, such as the PDB and nanobase.^107^ Like DNA origami, assembly of RNA origami can be achieved through multistrand assembly (i.e., small repeating structure motifs), or unimolecular folding (i.e., one long single-stranded RNA (ssRNA) folding into the target shape using intramolecular base pairing). The former strategy allows modular assembly, while the latter is akin to a preprogrammed chain that folds into a customized shape without additional staple strands. Here, we will review RNA nanostructure design-related methods based on two main approaches, including (1) building block assembly; and (2) ssRNA folding and self-assembly.

#### 2.6.1 RNA Building Blocks

The concept that a designed nanomachine could be built from RNA was mooted over two decades ago when the structure of packaging RNA (pRNA) from the bacteriophage phi29 DNA packaging motor was shown to contain a 3-way junction (3WJ) motif.^95^ As it is thermostable and allows further assembly with DNA or protein via the protruding motif, 3WJ can be used as a building block wherein each arm can be modified and can also be extended to form larger structures. This has been widely utilized, for example individual ssRNA strands carrying camptothecin and folic acid have been assembled into tumor-targeting 3WJs^108^ and 3WJs have been extended in to larger structures bearing anti-mRNA.^109^ Many assembled nanostructures have been demonstrated such as a 2D RNA triangle, square, pentamer, hexamer (Figure 4F), and arrays as well as 3D shapes including a tetrahedron, prism and dendrimers (see review^110^). The 3WJ motif has recently been further developed into a 4-way junction (4WJ-X) with even higher thermostability, resulting in a structure that remains intact even after 8 M urea treatment.^104^ In addition to naturally existing RNA motifs, SELEX has been used to iteratively select single strand RNA aptamers with high affinity to targets^97,98,111^ meaning it can be used for specific interaction or functions.

To expand structure complexity and size, “Tecto-RNA” was developed using rational design to introduce “struts” consisting of a double helices on individual RNA modular units, allowing for self-assembly into predefined three-dimensional structures by connecting the edges with loop-receptor interactions.^112^ The RNAJunction database documents experimentally verified RNA tertiary interaction motif structures, including RNA junction and kissing loops.^113,114^

Another tertiary interaction is the paranemic crossover (PX)^115^ derived from DNA assembly that allows two individual RNA units to connect via Watson–Crick base pairing with a minimum number of two crossovers spanning the major groove. As this crossover avoids denaturing module structures, it is reversible. NanoTiler, an automated approach, was later developed to computationally design self-assembling RNA structures from RNA 3-way junctions, bulges, and kissing loops.^116^ Most recently, the generative diffusion framework RFDpoly has been released from the Baker lab to aid in the design of *de novo* biopolymers such as RNA.^117^ While we cannot comment on the specific use cases of each novel software being released in the context of biological nanomachine development, it is important to appreciate the speed at which the field is currently developing.

#### 2.6.2 Single-Strand RNA Folding and Self-Assembly

The ability of single-strand RNA (ssRNA) to self-fold allows unimolecular assembly leading to formation of DNA-origami like programmable scaffolds but without a requirement for staple strands and represents a significant conceptual difference from previous “building block” style approaches.^103^ This strategy uses a single long RNA for unimolecular folding which can be produced isothermally via enzymatic machinery; either T7 or T3-RNA polymerase-mediated *in vitro* transcription in tubes, directly on mica,^118,119^ in synthetic cells,^98^ or alternatively using native transcription in living cells (e.g., bacteria^120^ or cell lines^119^). During transcription, self-folding takes place based on internal double strand helix formation and self-assembly into designed structures via selective base-pairing interactions, such as hairpins and kissing loops.^98,102^ These designed nanostructures have been shown to be scalable: from 350 nucleotides^97^ to several kilobases^100,119^ and replicable. Moreover, the demonstrated ssRNA scaffold sequences can be either completely synthetic^119^ or include fragments from native RNA transcript sequences.^97,105,121^ To expand the desired RNA folding characteristics for functionality, RNA structure prediction has been applied to generate RNA sequences to be incorporated into the customized ssRNA origami. This approach showcases not only the ability of RNA origami design to include functional capacities, but also the power of utilizing RNA structure prediction tools. The diversity of RNA structures alongside the difficulty in structural prediction presents a significant challenge when incorporating RNA within biological nanomachines compared to the previously discussed DNA-based approaches.^122^ To overcome this hurdle, current research has a strong focus on computational RNA structural prediction. While the specifics of this emerging field go beyond the scope of this guide it is important to acknowledge these new structural prediction tools as key factors in the development of biological nanomachine assembly.

To program single strand RNA nanostructures, several software tools have been developed, including RNA Origami Automated Design (ROAD),^100^ and ssOrigami.^119^ ROAD is a pipeline that allows users to express RNA origami via cotranscriptional folding. It designs the nanoscaffolds from a library of structure motifs, identifies potential folding issues and then performs sequence optimization. Overall, the automated pipeline takes account of various factors in RNA 3D modeling of structures, including thermodynamics, the folding pathway, sequence constraints and pseudoknot optimization. It also allows scaling up in size and expanding RNA modular diversity.

ssOrigami is an automated design tool for designing and synthesis of user-specified 3D shapes from single-stranded DNA or RNA. In much the same way as the previously discussed DNA origami technique, ssRNA origami utilizes a long single stranded scaffold strand to construct larger three-dimensional structures, without the need for additional staple strands. However, there is a dramatic increase in complexity during the design phase due to RNA’s proclivity to form secondary structures. More specifically, ssOrigami generates space-filling structures, adding the capacity to arrange surface features, scaffolding external molecular cargoes or customized spatial patterns. The software employs partially double-stranded RNA and parallel crossover cohesion to avoid knotting complexity during the folding process. Apart from ssRNA self-folding origami without auxiliary strands, DNA staple strands have also been used in ssRNA origami design, producing DNA/RNA hybrid origamis.^99^ In addition, employing RNA staple strands for ssRNA folding has been demonstrated^105^ resulting in designed nanostructures including a tube,^103^ nanobrick and 3D wireframe.^106^ Moreover, a mRNA scaffold has been shown to form a lantern shape with addition of only 2 circular RNA staples. The size of the lantern is defined while the shape is flexible, allowing balance in cell endocytosis and translation efficiency.^105^

To program ssRNA folded nanostructures with complementary DNA oligos, studies have used existing DNA origami tools to assist with the design,^123,124^ including cadnano (for details, see the DNA origami tool section and Table 1) and DNA Origami Sequence Design Algorithm for User-defined Structures (DAEDALUS, http://daedalus-dna-origami.org).^125^ To account for RNA:DNA duplex geometry, the routing of the desired mRNA-DNA origami was obtained using caDNAno and the 10.67 or 11 bp per turn (or 10.5 bp per turn for tightly bundled helices) should be applied in the design.^123,126^ Specifically, pyDAEDALUS, an open-source software to design origami based on surface-based representation of target 3D geometry, allows applying A-form dual-duplex wire-frame design rules for designing RNA scaffold with DNA staples.^127^ For top-down ssRNA origami sequence design, Tiamat has been used to design ssRNA sequences, avoiding repeated sequences.^119,120^

As this section has demonstrated, nucleic acids can be used not just as information storage materials but increasingly in engineering biology as building materials. This expansion of their capabilities reflects the increased understanding that their roles in nature (at least in the case of RNA) are wider than previously appreciated. Furthermore, we are now able to design structural and catalytic functionalities for nucleic acids beyond what is seen in nature. These are the types of roles which, in living systems, are dominated by proteins. It is no surprise then that designing new functionalities for proteins and even completely artificial functional protein structures is a major and growing area in bionanoscience research with new tools becoming available at a high rate. In the next section we will consider some of these tools.

## 3 Tools For Protein Nanotechnology

A core pillar of synthetic biology is the design of proteins with new or enhanced functions that can be readily produced in recombinant systems.^6^ In recent years, the protein design field has flourished, with the advent of many machine-learning (ML)-based tools that can facilitate the key steps required to create new-to-nature proteins. However prior to ML tools, many *de novo* proteins were designed using rational or knowledge-based engineering (see Figure 5A). Designed proteins are often inherently thermostable, express well and are often soluble. This makes them ideal for use in bionanotechnology, being straightforward to produce and use.

A simplified view of protein design^128^ breaks down the process into three major steps: backbone design, sequence generation, and structure validation (Figure 5B), each of which is considered in the following sections. Since the release of AlphaFold2 in 2021, the ability of researchers to predict the 3D fold of a given protein sequence with high confidence, along with the democratization of protein design and prediction tools has seen the protein design field greatly expand.^129^ Prior to AlphaFold2, many protein prediction programs^130^ centered around the use of template-based modeling to predict protein folds, however, this was limited by the availability of known structures with moderate-to-high sequence similarity to the query protein sequence, hence limiting protein prediction to the realm of “known folds.” Some *ab initio* modeling was possible with Rosetta (discussed further in Section 3.1.1) and other tools,^131^ and were used for filtering of designed protein folds, however with the success of AlphaFold at CASP14, these methods saw reduced use in favor of ML-based prediction methods.^129,132^

While advances in structure prediction have significantly increased our ability to validate designs, the recent breakthroughs in sequence generation and backbone design using a variety of ML methods have driven the advancement of *de novo* protein design, moving beyond the modification of known proteins or the combination of fragments of naturally occurring proteins. The significance of these developments is highlighted by the awarding of the Nobel prize in chemistry in 2024 for protein structure prediction and protein design.^133^ To date, protein design has been used to tackle a variety of challenges in biology, such as the design of novel enzymes,^134,135^ antibodies,^136^ protein cages,^137^ and protein lattices^138^ (see Figure 6 for some examples). The ability for these designs to react to their environments has started to be realized with conformational switching, triggered by ligand binding having been demonstrated.^139^ This makes it increasingly viable to generate nanomachines which can “sense” their environment and “react”.

**Figure 5:**
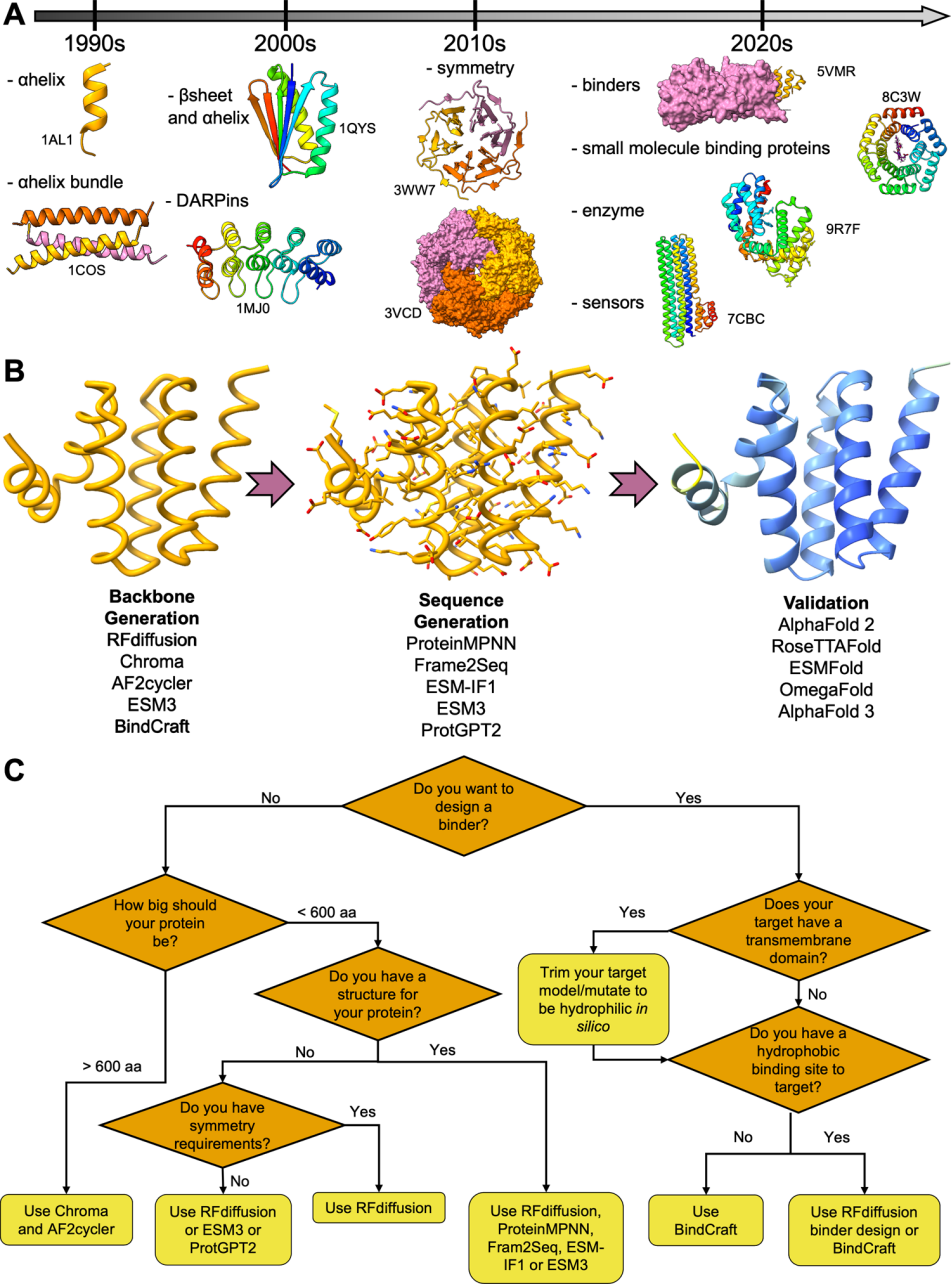
A generalized pipeline for basic protein design tasks. (**A**) A brief summary of the progress made to date in protein design. Starting in the 1990s with initially the ability to design single α helices (PDB 1AL1)^232^ and later bundles of α helices (PDB 1COS).^233^ Later more novel folds, with mixes of α helices and β sheets could be designed, like Top7 (PDB 1QYS)^141^ and designed ankyrin repeat proteins (DARPins, PDB 1MJ0).^234^ Later in the 2010s, symmetry could be utilized in protein design (PDB 3WW7, 3VCD).^235,236^ With ML designed tools now having been developed, protein design can be used for almost any conceivable challenge such as small molecule binding (PDB 8C3W),^237^ protein binding (PDB 5VMR),^238^ as novel enzymes (PDB 9R7F)^134^ and as sensors (PDB 7CBC).^239^ **(B**) Generic pipeline for protein design, with a selection of software that may be used for each section. (**C**) An example of some of the decisions that need to be made when selecting a pipeline/software for your chosen protein design task. A nonexhaustive list of considerations, biased by our experience.

**Figure 6:**
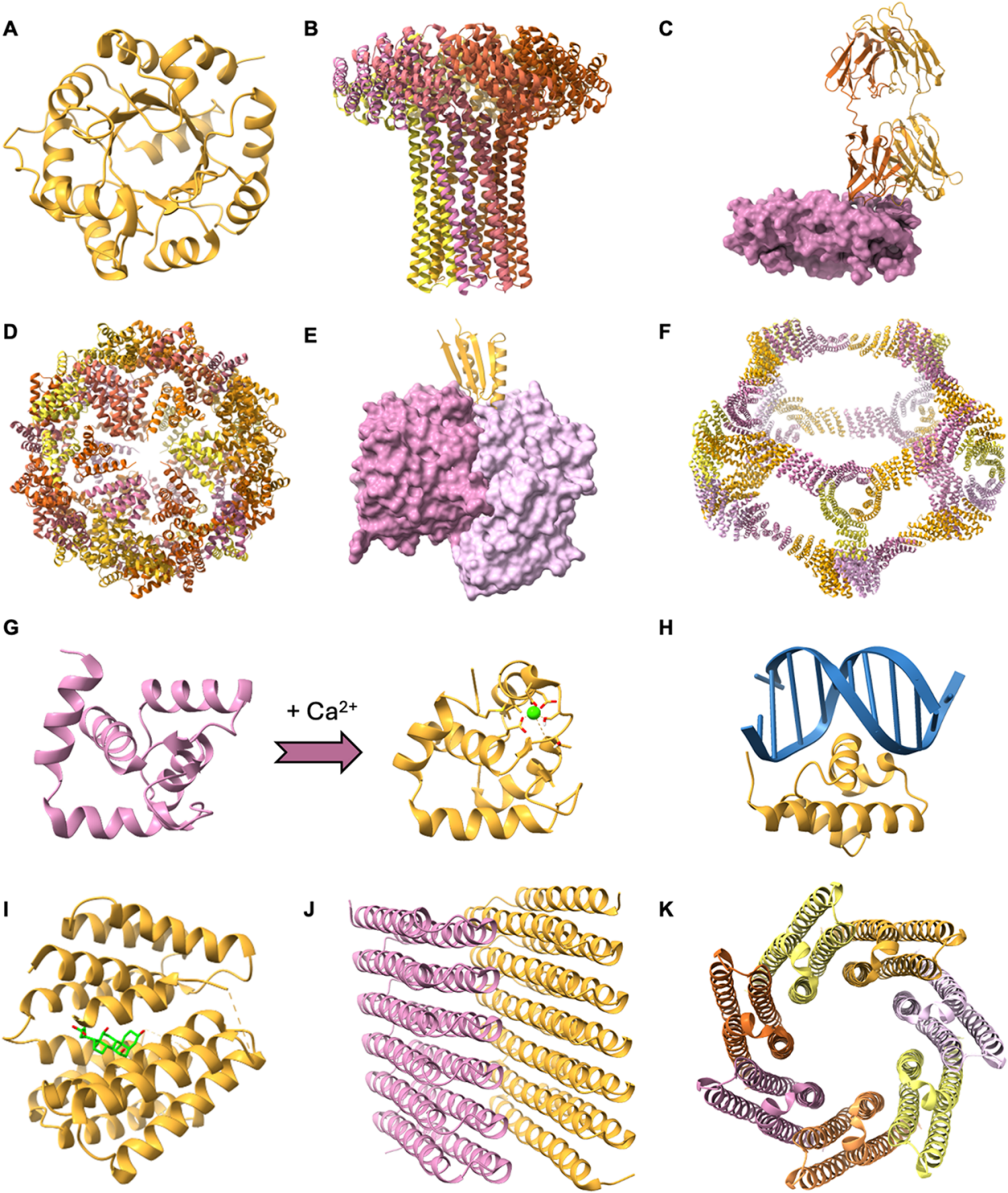
Examples of diverse designed proteins. (A) Structure of a designed retroaldol enzyme, designed using Rosetta (PDB 3NXF).^240^ (B) A designed 16-helix transmembrane porin (PDB 6U1S). (C) A designed antibody designed to bind IL-17A (PDB 5N7W).^241^ (D) A designed icosahedral homo-60mer protein cage (PDB 8F54).^242^ (E) A designed protein minibinder against integrin αvβ8 designed using Rosetta (PDB 8TCF).^243^ (F) A designed protein geometric lattice made of a bifaceted hetero-30mer with pseudo D5 symmetry (PDB 9DZE).^138^ (G) A designed protein which undergoes conformational change upon calcium binding, acting as a switch (PDB 9CIC and 9CIE).^171^ (H) A designed DNA binding protein (PDB 8TAC),^244^ (I) A designed cholic acid binding protein (PDB 8VEJ).^245^ (J) A protein designed to form a lattice for the control of biomineralization of calcium carbonate (PDB 8UGC).^246^ (K) A designed pore forming calcium channel (9DZW).^247^ Designed proteins are shown in cartoon representation, other proteins (i.e., binder targets) are shown in space filling representation. Small molecules shown as atoms in lime green.

In this section, we discuss some of the current state-of-the-art protein design tools, providing a standard workflow for the generation of new-to-nature proteins. We describe the tools available for every stage of *de novo* protein design, from protein backbone design through sequence generation, ending with protein prediction tools for the *in silico* validation of designed proteins to maximize the chances of producing proteins with the desired characteristics. An example of the decisions that a designer would take to choose software is shown in Figure 5C. We then discuss some of the available options for combining protein components of bionano devices together for the assembly of complete nanomachines. All the discussed tools are freely available, with details for where to access online tools and code being found in Table 3. We have selected key representative and pioneering tools for backbone design, sequence generation, and structure validation. Given the rapid growth of this field, an exhaustive survey of all available tools is beyond the scope of this review.

**Table 3:** A Summary of Selected Protein Design Software Tools

Software	Purpose	Ideal Use Case	Advantages	Drawbacks	Github Repository and Colab/server links
RosettaDesign	Backbone generation, sequence generation.	Design of very small proteins, or small sections of protein.	Physics-based. Rosetta toolkit in general is extremely useful for a wide variety of tasks.	Computationally expensive, very slow for large structures. Outperformed by ML-based methods.	https://github.com/RosettaCommons/rosetta
RFdiffusion	Backbone generation.	Design of most proteins. Best performance for proteins that are <600 amino acids long.	Extremely versatile, can be used for most protein design tasks. Highly successful and tested.	Computationally expensive, size limit, extensive *in vitro* screening required. Secondary structure bias for α helices.	https://github.com/RosettaCommons/RFdiffusion
					diffusion.ipynb - Colab
RFpeptides	Macrocyclic peptide binder design.	Design of macrocyclic binders against a target protein.	Allows for the design of macrocycles- greatly improved stability vs linear peptides.	Specific for macrocyclic peptide design.	https://github.com/Charlesjc-lab/RFdiffusion_RFpeptide
			Experimentally validated.		
CycleDesigner	Macrocyclic peptide binder design.	Design of macrocyclic binders against a target protein.	Allows for the design of macrocycles- greatly improved stability vs linear peptides.	Not validated *in vitro*.	https://github.com/hongliangduan/CycleDesigner
			Whole pipeline in one tool.	Specific for macrocyclic peptide design.	
RFdiffusion All Atom	Small molecule binder backbone generation.	Generation of proteins to bind a small molecule/DNA/RNA.	Can use small molecules/DNA/RNA *etc*. as well as protein.	Computationally expensive, size limit, extensive *in vitro* screening required.	https://github.com/baker-laboratory/rf_diffusion_all_atom
					RFdiffusion All Atom - Tamarind Online Tool
					https://colab.research.google.com/github/Graylab/DL4Proteins-notebooks/blob/main/notebooks/WS10_RFdiffusion_AllAtom.ipynb
RFdiffusion 2	Small molecule binder/enzyme backbone generation.	Generation of enzymes of a known activity or proteins to bind a known ligand.	Can design *de novo* enzymes using theozymes (theoretical transitions-state structure of an enzyme active site).	Computationally expensive, size limit, extensive *in vitro* screening required. Secondary structure bias for α helices.	https://github.com/RosettaCommons/RFdiffusion2
RFdiffusion 3	Backbone generation, small molecule binder/enzyme backbone generation, Nucleic acid binder backbone generation.	Generation of any protein backbone, especially when binding small molecule/nucleic acids.	Can use small molecules/DNA/RNA *etc*. as well as protein.	Computationally expensive, size limit, extensive *in vitro* screening required. Secondary structure bias for α helices.	https://github.com/RosettaCommons/foundry
Chroma	Backbone generation.	Design of large (>600 amino acid) proteins and protein complexes.	Computationally inexpensive, can handle large proteins.	Inaccurate backbones. (Although this problem is somewhat mitigated by AF2cycler). Secondary structure bias for α helices.	https://github.com/chroma-core/chroma
					ChromaDemo.ipynb - Colab
AF2Cycler	Backbone optimization.	Optimization of unrealistic backbones generated by Chroma	Can make backbones from Chroma more designable.	Only able to handle monomers.	https://github.com/sokrypton/ColabDesign/blob/main/af/examples/af2cycler.ipynb
ESM-3	Backbone generation, sequence generation.	Design of new versions of a target protein e.g., an enzyme.	Can do both backbone generation and sequence generation simultaneously, evolutionary based. Can generate a protein with a specified activity.	Evolutionary-based, so proteins may not necessarily be completely *de novo*.	https://github.com/evolutionaryscale/esm
					Evolutionary Scale · ESM3: Simulating 500 million years of evolution with a language model
BindCraft	Binder design.	Design of protein binders.	Highly successful binder design. Binders are ranked and evaluated by both Alphafold2 confidence metrics and Rosetta physics-based metrics.	Computationally Expensive.	https://github.com/martinpacesa/BindCraft
					BindCraft.ipynb - Colab
ProteinMPNN	Sequence generation.	Sequence optimization for almost any given protein.	Computationally inexpensive, fast, generates soluble, stable proteins. Integrated into many colab notebooks with structure generation tools.	Designed proteins using ProteinMPNN tend to be highly thermostable- this can be detrimental to some design tasks e.g., enzyme redesign where conformational changes are required.	https://github.com/dauparas/ProteinMPNN
					quickdemo.ipynb - Colab
					https://huggingface.co/spaces/simonduerr/ProteinMPNN
CAPE-MPNN	Sequence generation.	Generation of proteins with reduced immunogenicity.	Reduces the immunogenicity of designed proteins.	Only takes into account MHC Class 1 epitopes.	https://github.com/hcgasser/CAPE_MPNN
SolubleMPNN	Sequence generation.	Generation of soluble proteins.	Computationally inexpensive, fast, generates soluble, stable proteins. Integrated into many colab notebooks with structure generation tools.	Designed proteins using SolubleMPNN tend to be highly thermostable- this can be detrimental to some design tasks e.g., enzyme redesign where conformational changes are required.	See ProteinMPNN (integrated into vanilla ProteinMPNN)
ThermoMPNN	Thermostability evaluation	Evaluation of the outcome of single point mutations on thermostability.	Computationally inexpensive, fast.	Can only predict the outcome of single point mutations, indels or double point mutations.	https://github.com/Kuhlman-Lab/ThermoMPNN
					https://colab.research.google.com/drive/1OcT4eYwzxUFNlHNPk9_5uvxGNMVg3CFA#scrollTo=i06A5VI142NT
					https://github.com/Kuhlman-Lab/ThermoMPNN-D
					https://colab.research.google.com/github/Kuhlman-Lab/ThermoMPNN-D/blob/main/ThermoMPNN-D.ipynb
					https://colab.research.google.com/github/Kuhlman-Lab/ThermoMPNN-D/blob/ThermoMPNN-I/ThermoMPNN-I.ipynb
HyperMPNN	Sequence generation.	Generation of hyperthermophilic proteins.	Computationally inexpensive, fast, generates extremely thermostable proteins.	Only trained on bacterial sequences- could struggle with eukaryotic proteins.	https://github.com/meilerlab/HyperMPNN
LigandMPNN	Small molecule binder sequence generation.	Generation of proteins to bind a small molecule/DNA/RNA.	Can generate sequences which maintain binding to ligands/DNA/RNA *etc*.	For ligands that are rare or absent from the PDB, there will have been insufficient data in the training data set for reliable designs.	https://github.com/dauparas/LigandMPNN
					LigandMPNN_Colab.ipynb - Colab
Frame2Seq	Sequence generation.	Sequence optimization for almost any given protein.	Faster than ProteinMPNN, higher sequence recovery.	Less well experimentally validated than ProteinMPNN.	https://github.com/dakpinaroglu/Frame2seq
			Successful at multistate design.		Frame2seq ipynb - Colab
ESM-IF1	Sequence generation.	Sequence optimization, especially for a protein with multiple conformations.	Can design a protein with multiple defined conformations.	No experimental validation.	https://github.com/LBC-LNBio/ESMIFDesign/
ProtGPT2	Sequence generation.	Generation of proteins in remote parts of the protein structure/sequence space.	Can access protein sequence space that is unexplored by diffusion models- no secondary structure bias.	Cannot so easily control what will be generated.	https://github.com/TeletcheaLab/protGPT2

### 3.1 Backbone Design

Often, the first problem to be tackled in a protein design pipeline is to generate a novel backbone suitable for a required function. This backbone may need to conserve certain structural motifs or elements in a template protein, may be completely unconstrained, or may be required to bind to a target protein. There have been several different approaches developed to address the problem of generating novel protein backbones (Table 3). These may simply generate a backbone alone or may also simultaneously generate a sequence, although sequence generation methods, described in Section 3.2 are often still used to redesign these sequences. The choice of design tool will be affected by the problem to be solved by the designer and so there is no “silver bullet” pipeline which should always be used. We will briefly discuss some of the most recent and broadly applicable tools to be developed, highlighting their strengths and limitations.

#### 3.1.1 RosettaDesign

Before the advent of ML-based tools, RosettaDesign was extensively used for protein design. RosettaDesign is a module within the larger Rosetta software suite, specifically tailored for protein design.^140^ The Rosetta software suite provides a variety of diverse tools that can be used for important design steps, such as structure energy minimization, sequence generation, structure prediction evaluation and protein–protein and protein–ligand docking etc. Rosetta especially thrives when incorporating nonstandard amino acids and chemical modifications into these designs which is not possible with many ML-based methods. RosettaDesign operates by assembling a protein backbone from a large library of short fragments. This assembly process is guided by a Monte Carlo method, which optimizes the backbone in favor of low-energy, stable configurations by utilizing data from known protein structures and physical constraints (electrostatics, van der Waals interactions etc.) to score protein conformation and sequence. Once the initial backbone is designed, the model performs iterative perturbation and evaluation for lowest-energy conformations.

RosettaDesign is physics-based, utilizing models of energetics calculations, making each design optimized for folding and stability regardless of their novelty. Utilizing energetics calculations to determine an optimal structure makes this method very robust, but much more computationally intensive than ML-based tools (discussed below). Therefore, RosettaDesign is best used for the design of small, highly novel artificial proteins that are not likely to possess analogues found in nature (although symmetry can be exploited to allow for the generation of larger architectures) or as an optimization tool, particularly for binding sites and side-chain interactions.^141^ Indeed, RosettaDesign has been demonstrated to be a powerful tool to guide designs and optimize binding and foldability, as exemplified by its use to create an artificial luciferase protein.^142^ Beyond RosettaDesign, the Rosetta suite offers an exceptional means to assess the quality of designs and should regularly be used in conjunction with other design tools to energy minimize models of designs, and evaluate their energetic feasibility, and is indeed integrated into multiple different workflows, such as the BindCraft pipeline (outlined below).

#### 3.1.2 RFdiffusion

Developed by the Baker Lab, RFdiffusion^128^ designs protein backbones using a generative diffusion process using the RoseTTAFold (RF) as a pretrained model that has “learnt” protein folding (RoseTTAFold is discussed in detail in Section 3.3.3). Noised, randomized residues represented only by backbone atoms are denoised using this RF model into realistic protein backbones in an iterative process. By utilizing deep learning, RFdiffusion is highly computationally efficient. Because the noising and denoising is conditional, RFdiffusion can be used for almost any protein design task, such as designing hetero- or homo-oligomers, symmetrical oligomers and generating *de novo* protein binders. Protein binders have successfully been designed against a variety of different protein targets to date, including more challenging targets such as intrinsically disordered proteins (IDPs) and snake venoms.^143,144^

RFdiffusion also supports partial diffusion, where the user can supply part of a protein structure (a fold, scaffold or binding motif), which is noised and then denoised to generate a compatible completed backbone. This allows the redesign of a protein with a known fold and has been used to improve the quality of known protein binders.^145^ Alternatively, fixed functional motifs or active sites can be used as templates, with the surrounding protein structure being generated *de novo*. This enables the generation of proteins with similar function to known enzymes but with entirely novel structural scaffolds.

The ability to generate protein binders to a given target is arguably one of the most powerful functions of RFdiffusion.^128^ Binder generation exploits surface exposed hydrophobic residue hotspots to define binder interaction sites on the target protein, however reliance on these residues can mean that some targets are difficult to design binders against. Additionally, RFdiffusion designs (in common with those generated by most generative models) tend to overfavor α-helical structures, a bias that can be somewhat mitigated with an alternative “β model” that improves the generation of β-strand/sheet-containing proteins. Moreover, there is a soft size limit of approximately 600 amino acids. While RFdiffusion can be used to make designs larger than this, model performance begins to become less reliable i.e., the RMSD between the RFdiffusion derived backbone and the AlphaFold prediction begins to increase, meaning that they diverge.^146^ Notably, larger, capsid-sized backbones can be generated using RFdiffusion, though it achieves these scales through the design of small repeating units that self-assemble into large protein cages.^147^

Since RFdiffusion is a deep learning model, it can be repurposed with additional training to design certain classes of proteins. For instance, Bennett et al. partially retrained the model on antibody complexes to design *de novo* single domain antibodies.^148^ A pair of tools, CycleDesigner and RFpeptides, use modified versions of RFdiffuison for the design of cyclic peptide binders.^149,150^ Finally, the recent release of RFdiffusion2 expands the repertoire from just amino acids to being able to incorporate noncanonical elements, such as small- molecule ligands.^151^ RFdiffusion3 then further expands to be able to incorporate nonprotein molecules and atoms (nucleic acids, small molecules, metal ions etc.), allowing the design of proteins with new and diverse ligand specificity beyond protein–protein interactions.^152^

RFdiffusion is emerging as a workhorse for protein design, with RFdiffusion backbone design, ProteinMPNN sequence generation and AlphaFold2 validation forming a standard workflow for protein design from backbone to validation.

#### 3.1.3 Chroma

Chroma^153^ is a diffusion-based model that utilizes a graph neural network (GNN) architecture trained on larger proteins than those typically used in other tools, making it well suited for designing proteins exceeding 600 amino acids. In Chroma, each amino acid is represented as a node in a graph, with interactions between residues encoded as edges. This basic representation allows Chroma to efficiently model residue–residue interactions, making it relatively computationally inexpensive when generating large proteins. Chroma also employs a low-temperature sampling strategy which, while reducing diversity, improves the quality of the backbone designs and is still capable of generating highly novel proteins. The structural novelty achieved by Chroma is not limited to large-scale assemblies and extends to a wide range of possible structural features. Chroma is designed to be customizable, utilizing heuristic classifiers (conditioners), which allow the incorporation of symmetry, functional substructures (e.g., active sites), user-defined shapes, structural semantics, and can even interpret natural-language prompts. Modifying these conditions requires some programming experience, however guidance is available in the Chroma documentation. The use of a GNN by Chroma facilitates modeling of proteins far larger (up to capsid-size proteins), however, structural detail is lost in this process, making other models offering atomistic models a better solution for designing smaller proteins. This capacity for large-scale backbone generation is primarily drive by the Chroma network’s sparsity, which significantly reduces the memory required for extensive backbones. Consequently, Chroma may lack the precision of other tools for some design campaigns.

It is important to note that Chroma-generated backbones are often not physically realistic in isolation. Therefore, additional steps such as structure relaxation, energy minimization, or Alphafold2 refinement may be required before sequence design. When working with larger protein designs, the AF2cycler pipeline (Table 3), which uses iterative Alphafold2 hallucination and refinement, can improve the quality of Chroma backbones.^154^

Hallucination emerged as a prominent protein design technique following the release of AlphaFold2, though it was largely superseded by RFdiffusion as the standard for backbone generation. The process begins by generating a randomized protein sequence and predicting its structure via AlphaFold; a loss function is then applied through iterative mutations until the sequence converges on a structure that matches the desired fold.^155^ This method proved versatile for inpainting (filling gaps in incomplete structure), de novo backbone generation, and binder design.^156,157^ Ultimately, diffusion models overtook hallucination in widespread use due to their significantly higher success rate and computational efficiency.^146^

#### 3.1.4 BindCraft

BindCraft^158^ is a protein binder design pipeline that leverages AlphaFold2-based protein hallucination with gradient backpropagation to generate novel binders (Alphafold2 is discussed in detail in Section 3.3). It has boasted high success rates for designing binders against a variety of different protein targets. This pipeline integrates various models throughout the design process, encompassing structure generation, sequence development, and structural validation, including AlphaFold2, ProteinMPNN and Rosetta. BindCraft is specialized for binder design, and the developing authors report success rates ranging from 10 to 100% for experimentally tested targets.^159^

The first step of the design process combines both backbone generation and sequence generation via AlphaFold2 hallucination. A desired protein target is selected, and residues or regions can be chosen as specific epitopes, along with the length range of the intended binder. The model then generates an initial random sequence for the binder, and Alphafold2 multimer is employed to predict the potential complex formed by the target and binder. Because Alphafold2-multimer is trained on protein complexes, backpropagation through its confidence metrics allows for the optimization of realistic protein–protein interfaces. By comodeling the binder and the target, BindCraft can account for the alternate conformational states of proteins, unlike many preceding binder design tools that treated the input target protein as a rigid body. This approach acknowledges that protein–protein interactions often depend on conformational states that are not captured by the apo structure, improving the likelihood of successful binder design.

Interface residues are then fixed, and the remaining sequence is optimized using SolubleMPNN, a version of ProteinMPNN (discussed in detail in Section 3.2) trained specifically on soluble proteins. While the interface (and therefore binding ability) is unchanged here, the overall stability and solubility of the protein is likely to be improved. After backbone and sequence design, structural validation is performed with Alphafold2-monomer, which is trained on single-chain protein sequences and avoids artificial reinforcement of binding contacts. Designs are automatically filtered based on AlphaFold- and ProteinMPNN-based confidence scores, along with Rosetta-based energy and interface metrics. The program runs until the specified number of designs passing confidence scores and metrics are generated.

Due to repeated Alphafold2 inference and backpropagation, BindCraft is a computationally intensive, often requiring trimmed or simplified target structures as inputs. However, despite being computationally demanding, BindCraft is an exceptional user-friendly software that exhibits several desirable design features: A high proportion of the designed proteins can be expressed and purified as soluble proteins that retain binding activity due to implementation of rigorous *in silico* screening; It supports binder design to both known and previously unidentified binding epitopes, and while BindCraft performs best when hydrophobic surfaces are present it is not strictly dependent on them.

If the goal of the design process is to generate small protein binders to defined proteins/epitopes, and the user has sufficient computing time and power, it is our opinion that BindCraft is an excellent choice. As with most protein design tasks, however, success is highly dependent on the target.

### 3.2 Sequence Generation

Once a backbone has been generated, the next step in the design process is to identify a sequence that will reliably fold into that structure. The input backbone may originate from the *de novo* backbone generation tools or alternatively can be used to redesign known protein structures. This process of determining a compatible sequence from a fixed structure is commonly referred to as inverse folding. As with backbone generation, numerous tools have been developed to complete this task. Below, we describe several representative approaches.

#### 3.2.1 ProteinMPNN

ProteinMPNN^160^ is one of the most extensively used tools for sequence generation in protein design. It is based on a message-passing neural network (MPNN), a type of graph neural network in which atoms are represented as nodes and their structural relationships as edges. In ProteinMPNN, each node corresponds to an atom in an amino acid, and edges encode geometric relationships (e.g., interatom distances) derived from a fixed input backbone. The network iteratively passes information (messages) between neighboring residues to learn the likelihood of an amino acid for a given position. After several iterations of message passing, the model outputs a probability distribution over amino acids at each position in the protein. A sequence is then sampled from the probability distribution, depending on the set temperature. Because it was trained on experimentally determined structures from the PDB, ProteinMPNN tends to generate sequences that are highly thermostable and soluble. This means that enzymes can be improved for increased stability, without a cost to activity, as demonstrated in the improvement of both tobacco etch virus (TEV) protease and myoglobin.^161^

ProteinMPNN is highly versatile, allowing for the design of homo-oligomers, binder generation, selective fixing of residues, and enzyme redesign by preserving key catalytic residues while varying those which are not required for activity. Users can adjust the sampling temperature to control sequence diversity and exclude certain amino acids (e.g., cysteine) if desired.

ProteinMPNN complements tools like RFdiffusion, which produce backbone-only structures with placeholder resides (typically glycines), by providing compatible amino acid sequences. It is fast and computationally inexpensive, allowing for hundreds of different sequences to be generated from a single backbone in minutes with very little computational power. ProteinMPNN can also be applied to redesign natural proteins^161^ while retaining key residues enabling improved solubility, stability and/or expression. It can be used to complement backbone generation tools, such as Chroma, and is also integrated into the BindCraft pipeline as well as the Google Colab notebooks for RFdiffusion and BindCraft.^158^ A huggingface space is available for standalone ProteinMPNN, with integrated Alphafold2 validation.^162^

To date, several ProteinMPNN variants have been developed for specialized applications, each trained on custom data sets; key examples are listed as follows: CAPE-MPNN enables control over the immunogencity of the designed proteins.^163^ SolubleMPNN, trained exclusively on soluble proteins, is designed to improve solubility;^164^ this was used to design soluble homologues of membrane proteins by redesigning the hydrophobic, lipid interfacing surfaces, improving expression, purification and characterization.^164^ ThermoMPNN, trained on thermophilic protein sequences, predicts changes in protein stability resulting from point mutations. It uses the same messenger-passing framework as ProteinMPNN and is well suited for rational optimization in cases of proteins, where only limited mutations are permitted (such as in the design of new vaccines).^165^ HyperMPNN was trained on the structures of hyperthermophilic proteins and will give protein sequences which are more thermostable than those produced by standard ProteinMPNN.^166^ LigandMPNN is an all-atom version of MPNN that can also handle ligand atoms, which can be utilized for enzyme design and for the design of small molecule-binding proteins while NA-MPNN can be used the design of both RNA sequences and nucleic acid binding proteins.^167,168^

A recent advancement in the application of ProteinMPNN is the generation of multistate proteins capable of conformation switching. This capability is particularly promising for the development of nanomachines, as these structural transitions can be engineered to trigger in response to specific environmental stimuli, such as ion or ligand binding, temperature or pH changes.^139,169^

#### 3.2.2 Frame2Seq

Frame2Seq is an inverse folding model, like ProteinMPNN, that generates sequences compatible with a fixed protein backbone. It has demonstrated fast sequence generation and tends to produce designs with high solubility and expression. Frame2Seq offers fast inference and excellent sequence recovery (a higher percentage identity between designed sequences and native input sequences).^170^ Additionally, Frame2Seq excels at multistate design- in which proteins have conformational switching. For example, the Kortemme lab recently demonstrated the design of a dynamic protein that locked to a single conformation upon ion binding, using Frame2Seq.^171^

#### 3.2.3 ESM-IF1

ESM-IF1 is another inverse folding model that predicts protein sequences based on an input backbone structure.^172^ Trained on over 12 million Alphafold2 predicted structures, ESM-IF1 can also infill missing regions within an input backbone. Notably, it can be conditioned to generate sequences with multiple conformation states, which may be valuable in applications where conformational switching underpins function, such as in synthetic nanomachines or allosteric proteins.

#### 3.2.4 ProtGPT2

ProtGPT2 is a protein design model based on the GPT-2 language model architecture. It was trained on a large data set of natural protein sequences, using next-token prediction to generate novel sequences one residue at a time.^173^ Although the sequences generated by ProtGPT2 are not associated with specific 3D structures, as the case for backbone generation tools, the model expands the accessible sequence space beyond that found in natural protein databases. To enable rational design of backbones using ProtGPT2, generated sequences can be structurally evaluated using Alphafold2, and subsequently compared to known protein structures using FoldSeek to identify those likely to adopt a desired fold.^174^ This approach also holds promise for generating completely novel proteins in an unsupervised and unbiased manner, without reliance on predefined structural templates. A variation of ProtGPT2, ZymCTRL, has been developed for the design of new enzymes of a specified enzyme class.^175^

#### 3.2.5 ESM-3

ESM-3 is a large language model (LLM) for protein design that generates amino acid sequences and predicts their structures, enabling the generation of novel proteins.^176^ While it does not model the physical constraints of proteins explicitly, it has learnt the underlying ‘grammar’ of protein sequence, structure, and function through large-scale evolutionary training. ESM-3 tokenizes amino acid sequences and was trained using a masked language model to predict missing residues based on the surrounding sequence context. It was then trained on hundreds of millions of protein sequences from diverse evolutionary lineages, enabling the learning of conserved relationships between protein structure and function.

Because ESM-3 was trained on a great variety of protein sequences, it can generate new proteins based on statistical patterns learned from its evolutionary context. Although often biased toward known sequence and structure families, ESM is less computationally intensive that other diffusion models and can generate novel proteins that have a basis in evolutionary data. To validate ESM-3, the model was used to generate a designed variant of green fluorescent protein (GFP) with only 58% identity to its closest known homologue, and was experimentally demonstrated to retain fluorescence.^176^ Additionally, ESM-3 outputs can be integrated into geometric deep learning models, which explicitly graph residue interactions in a protein structure in 3D space to facilitate design, to improve structure prediction and design performance.^177^

### 3.3 Validation

A wide array of protein structure validation tools are available, each providing various metrics to help evaluate model quality. Details of these can be found in Table 4, and a list of some of the available software can be found in Table 5.

**Table 4:** Parameters Used to Describe Predicted Protein Structure

Abbreviation	Full name	Meaning
RMSD	Root Mean Square Deviation	A measure of distance between corresponding atoms in two superimposed structures. The lower the value the closer those atoms are.
TM-score	Template Modeling score	A measure of similarity between two protein structures not depending on proteins length.
		TM-score takes values between 0 and 1, where 1 represents perfect match of two structures.
		Values <0.2 suggest there is no similarity between structures.
		Values >0.5 suggest proteins have similar topology.
pLDDT	Predicted Local Distance Difference Test	Score which evaluates local distance differences of atoms in a model.
		Measures confidence of the model in scale 0–100. Scores above 90 are expected to be modeled with high accuracy. Values between 70 and 90 are modeled well. Values below 70 should be treated with caution and below 50 not interpreted at all.
		For ligand atom test considers only ligand atoms.
pAE	Predicted Aligned Error	Parameter which estimates error of the predicted position in the context of predicted structure.
		Higher values mean higher predicted error and lower confidence.
pTM	Predicted Template Modeling	Measures accuracy of the predicted model.
		Value above 0.5 means predicted fold might be like actual structure.
ipTM	Interface Predicted Template Modeling	Measures accuracy of the predicted subunits positions in the predicted complex.
		Values >0.8 mean high confidence interface predictions. Between 0.6 and 0.8 interfaces are not sure. Below 0.6 prediction failed.

**Table 5:** Summary of Software Properties and Limitations

Software name	Github Repository and Colab/server links	Input length limitation	Multimers?	Ligands available?	Advantages
Alphafold2	GitHub - googledeepmind/ alphafold: Open source code for Alphafold2, https://github.com/google-deepmind/alphafold	No, but long sequences increase prediction time	Yes	No	No size limitation
	AlphaFold2.ipynb-Colab				
RoseTTAFold	GitHub-baker-laboratory/RoseTTAFold-All-Atom	>27 aa, <1400 aa	Yes	No	High precision, slower than other tools
	robetta.bakerlab.org				
OmegaFold	GitHub-HeliXonProtein/OmegaFold: OmegaFold Release Code, https://github.com/HeliXonProtein/OmegaFold	Up to 4096 aa	Yes	No	Does not use MSA—better prediction for nonhomology sequences, rapid structure prediction
	omegafold.ipynb-Colab				
ESMFold	GitHub-facebookresearch/esm: Evolutionary Scale Modeling (esm): Pretrained language models for proteins, https://github.com/facebookresearch/esm	Sequences above 1200 aa are very computationally expensive	No	No	Rapid structure prediction
	Resources - ESM Metagenomic Atlas, https://esmatlas.com/resources?action=fold	400 aa for the online server			
AlphaFold 3	GitHub-google-deepmind/alphafold3: AlphaFold 3 inference pipeline, https://github.com/google-deepmind/alphafold3	5000 aa	Yes	Yes: DNA, RNA, ligands and ions (only small library available in online server)	Additional ligands/compounds available for modeling
	AlphaFold Server, https://alphafoldserver.com/				
Chai-1	GitHub-chaidiscovery/chai-lab: Chai-1, SOTA model for biomolecular structure prediction, https://github.com/chaidiscovery/chai-lab	2048 aa	Yes	Yes: DNA, RNA, ligands, ions	Additional ligands/compounds available for modeling
	Chai Discovery, https://lab.chaidiscovery.com/auth/login?callbackUrl=https%3A%2F%2Flab.chaidiscovery.com%2Fdashboard				Can add user-defined restraints to improve the quality of the prediction of complexes.
	https://huggingface.co/chaidiscovery/chai-1				
Boltz-2	GitHub-jwohlwend/boltz: Official repository for the Boltz biomolecular interaction models, https://github.com/jwohlwend/boltz	No, but long sequences increase prediction time.	Yes	Yes: DNA, RNA, ligands, ions	Additional ligands/compounds available for modeling.
					Can predict binding affinity for small molecules.

#### 3.3.1 I-TASSER

One of the earliest tools available for protein structure prediction was an automated structure prediction server by the Zhang Lab called I-TASSER.^130^ I-TASSER constructs models by identifying structural fragments from the PDB, which are then assembled into full-length proteins. Regions of the protein lacking suitable templates, loops or disordered segments, are modeled using *ab initio* methods. Energy of generated models is evaluated, and low energy candidates are selected for further refinement to reduce steric clashes and improve global topology. Hydrogens are added in the final optimization step to assist with clash detection and hydrogen bond refinement. Since its launch, I-TASSER has been expanded to support multidomain proteins^130^ and incorporates deep learning-based improvements through the C-I-TASSER pipeline, and more recently D-I-TASSER, which performed extremely well in CASP14 and CASP15.^178,179^

#### 3.3.2 Alphafold2

AlphaFold, developed by DeepMind, revolutionized protein structure prediction. The original AlphaFold was released in 2020, followed by Alphafold2 in 2021, which significantly advanced prediction accuracy.^180^ The core innovation of Alphafold2 was a novel end-to-end structure prediction pipeline powered by deep neural networks. The input amino acid sequence is first processed through a multiple sequence alignment (MSA), capturing evolutionary relationships across homologous proteins. The resulting features are analyzed by a deep neural network trained on PDB structures to predict inter-residue distances and orientations. These predictions are used to construct probability distributions over pairwise atomic distances between residues. Alphafold2 refines backbone geometry using gradient descent on learned structure-based potentials, minimizing an internal loss function that favors physically plausible and accurate models. The resulting predicted models typically show low RMSD and high TM-scores when compared to experimental data. A key innovation in Alphafold2 was the introduction of the Evoformer module, a neural network architecture that jointly processes MSA and pairwise representations. It integrates information from MSAs and pairwise residue features, enabling accurate prediction of inter-residue distances and orientations. The resulting 3D models can optionally be further refined using an AMBER force field to resolve minor clashes and improve local stereochemistry.

#### 3.3.3 RoseTTAFold

RoseTTAFold was released by the Baker lab in 2021. While inspired by AlphaFold it introduced a distinct multitrack neural network architecture that jointly models sequence, distance and coordinate information.^181^ The model simultaneously processes three types of information: 1D sequence alignment, 2D inter-residue distance maps, 3D coordinate representations. Information flows between all three levels via attention mechanisms, allowing the neural network to learn relationships between sequences, structural constraints and 3D structure. The learned representations from the 1D and 2D tracks inform the 3D track, which predicts the backbone coordinates. The model was trained on experimentally determined structures from the PDB, with a maximum length of 260 amino acids during training.

#### 3.3.4 OmegaFold

OmegaFold is another deep learning-based tool for protein structure prediction developed by Helixon.^182^ Like RoseTTAFold and Alphafold2, OmegaFold is trained on experimentally determined structures from the PDB. The main difference is that OmegaFold does not rely on MSA or evolutionary data as inputs. However, its structural accuracy is generally lower than that of Alphafold2 for predictions of well-characterized proteins.

#### 3.3.5 ESMfold

ESMfold is a protein structure prediction model developed by Meta AI in 2023.^183^ It utilizes an evolutionary-scale transformer language model (ESM-2), trained on millions of protein sequences from UniRef50, to directly predict the 3D structure of a proteins without need for MSA. As for OmegaFold, this simplifies the prediction process leading to much faster interference times. Its fast run times make it suitable for metagenomic scale structure prediction and large-scale data analysis. It is worth noting that because ESMfold and OmegaFold do not rely on MSAs, they are less affected by biases related to evolutionary conservation, making them more suitable for predicting structures of orphan or synthetic sequences. For high-throughput structural screening with limited computational resources, it may be advisible to use OmegaFold for a rapid validation, followed by ESMFold or Alphafold2/3 to further validate the top candidates.

#### 3.3.6 AlphaFold 3

AlphaFold 3 was published in 2024 by DeepMind.^184^ Unlike its predecessor, AlphaFold 3 is a unified diffusion-based generative model that incorporates a 3D equivariant neural network backbone and guided energy functions. It can predict structures of proteins and their interactions with a wide range of binding partners, including nucleic acids, small molecules, ions and post-translational modifications. However, predictions are currently limited to predefined libraries of small molecules, ions and modifications. The model also supports accurate RNA structure prediction, achieving performance comparable to RNA-specific methods. It outperforms other state-of-the-art tools in predicting protein–ligand complexes, including protein–DNA and antibody–antigen interactions. As with other diffusion-based models, AlphaFold 3 has propensity to introduce α-helical content in regions of low confidence; users should therefore avoid overinterpreting low confidence predictions.

Several open source reimplementations of AlphaFold 3 were developed prior to the release of the AlphaFold 3 code to improve accessibility to the wider community and extend functionality. Before the code was released, users could only access via the AlphaFold3 server, which is limited to a certain number of predictions per day and only specific defined ligands and modifications. Chai-1 (developed by the Wu Lab)^184^ Boltz-2 (developed by the Marks and Madani Laboratories)^185^ are two independently trained models that broadly recapitulate AlphaFold 3′s architecture and capabilities. Boltz-2 includes additional modules for confidence estimation, ligand placement, and epitope targeting along with prediction of ligand binding strength. Chai-1 includes the ability to input user-specified restraints based on experimental results to improve the quality of complex prediction.

#### 3.3.7 Transitioning from Computational Design to Experimental Validation

In this section, we give representative pipelines that serve as exemplars to signpost new users toward the foundational tools of modern protein design. Given that this is an exceptionally fast-moving field, it is likely that groundbreaking tools have been released during the writing and submission of this review. Consequently, we have focused on a limited number of core pieces of software cognizant that many specialized tools available for various stages of the protein design process remain beyond the scope of this work.

Due to the inherent uncertainties in protein folding, it is standard practice to apply rigorous *in silico* filtering such as evaluating predicted folding confidence through metrics such as predicted local distance different test (pLDDT) and structural similarity (RMSD), to narrow down thousands of computationally design candidates to a manageable subset for experimental testing. Even after this filtering, ML-designed proteins may still exhibit conventional challenges including poor expression and solubility, misfolding, or unintended oligomerization. For this reason, it is often a necessity to test multiple designs per design campaign even after strict *in silico* filtering. For the development of protein-based nanomachines, the final challenge is the assembly process, requiring methods to integrate distinct modules into a functional whole. In the following section we highlight key methods of the modification of proteins for the assembly of large-scale nanomachines.

### 3.4 Modification Tools on Protein-Based Nanomachines

There are multiple ways to combine modules in protein-based biological nanomachines. These methods can broadly be classified as chemical, enzymatic, spontaneous, and orthogonal. Each method uses a different approach, but in general, the chemical, enzymatic, spontaneous approaches directly modify the purified recombinant proteins, while orthogonal methods require additional steps during protein expression within cells.

Chemical reactions used to modify proteins often overlap with those used for DNA modifications (see Section 2.4.1 and Table 2). For example, Michael-type addition can be used to attach chemical compounds to thiol groups on free cysteines. This allows the conjugation of maleimide-containing compounds (e.g., fluorophores, lipids, DNA) to available cysteine residues. Another widely used chemical strategy targets amine groups found on lysine residues and the N-terminus of proteins. These are modified using activated esters (e.g., NHS esters), enabling similar conjugations as the maleimide–thiol reaction.

Chemical methods are, however, generally irreversible and nonspecific, meaning they target all accessible residues indiscriminately. While buried or structurally constrained residues are less likely to be modified, this lack of specificity can result in multiple unwanted decorations on the protein, which may be problematic when only a single modification is desired. Additionally, many chemical methods require reaction conditions which are compatible with nucleic acids, but not necessarily compatible with proteins due to being too harsh. Notably, commercially available compounds can link into pathways, methods mentioned in Table 2. For example, proteins can be initially modified with NHS-azide or NHS-DBCO (dibenzocyclooctyne) linkers, which later can be used for copper free cycloaddition (click chemistry), as detailed in Table 2.

Several enzymatic methods have also been developed. These rely on the use of naturally occurring enzymes that recognize specific peptide sequences and catalyze the formation of covalent bonds between two peptides (or chemical molecules). These enzymes are often engineered to enhance the performance or precision of the reaction (optimization of interacting peptide tag sequence). Peptide tags might be introduced by genetic fusion to the interacting partners or via chemical methods. Compared to chemical methods, enzymatic approaches offer greater specificity of protein modification (one modification per tag) and are also require milder conditions which are more compatible with proteins.

Examples of enzymes used for attachment to protein components include Sortase A (SrtA),^186–188^ surfactin phosphopantetheinyl transferase (Sfp),^189,190^ protein biotinylation by biotin ligase (BirA),^191^ and SnoopLigase^192^ described in detail in Table 6. Each enzymatic method has unique advantages and limitations and must be carefully optimized for the intended application. It is worth noting that these tags often function best when located at the N- or C-terminus of the protein but can also be effective when placed in accessible internal loops. However, reaction conditions need to be optimized for each specific reaction.

**Table 6:** Enzymatic, Spontaneous, and Noncovalent Modification Methods

	Sortase-mediated ligation (sortagging)	Sfp mediated ligation	Protein biotinylation	SnoopLigase:SnoopTag:DogTag	SpyTag:SpyCatcher	HisTag
Enzyme used	Sortase A (SrtA)^186–188^	Surfactin phosphopantetheinyl transferase (Sfp)^189,190^	biotin ligase (BirA)^191^	SnoopLigase^192^	none	none
Reaction type	Enzymatic transpeptidation	Enzymatic phosphopantetheinylation	Enzymatic biotinylation	Enzymatic isopeptide bond formation	Spontaneous isopeptide bond formation	Metal ion affinity
Bond formation	Covalent bond	Covalent bond	Covalent between AviTag-biotin	Covalent bond	Covalent bond	Noncovalent interaction
Partner A requirements	LPXTG-X_n_ peptide on the C-terminus	ybbR tag (13aa):	AviTag (15aa):	SnoopTagJr (12 aa):	SpyCatcher (116 aa)	Between 6 and 10 His residues
		DSLEFIASKLA	GLNDIFEAQKIEWHE	KLGSIEFIKVNK		
		Can be present on any end of protein, also internally but it has to be available for Sfp (efficiency might decrease)	Can be present on any end of protein, also internally but it has to be available for BirA (efficiency might decrease)			
Partner B requirements	Polyglycine tag G_n_ on N-terminus	Coenzyme A (CoA) modified partner	Biotin modified partner	DogTag (23 aa):	SpyTag (13 aa):	Nitrilotriacetic acid (NTA) modified partner
				DIPATYEFTDGKHYITNEPIPPK	AHIVMVDAYKPTK	
Pros	Small tags on both partners; Polyglycine can be present in internal loop but needs to be optimized	CoA can be easily attached to DNA or peptides during their production; Small protein tag	Library of commercially available biotinylated compounds; Can be used *in vivo* and *in vitro*	Small protein tag;	Spontaneous reaction, no enzyme needed;	Small protein tag (widely used); wide library of chemicals conjugated with NTA.
				High modification yield	Fast; Both tags can be present on either N- or C- terminus and in internal loop	
Cons	SrtA reaction might be reversible; reaction must be done in conditions suitable for SrtA	Requires magnesium ions; decoration may need to be optimized; reaction must be done in conditions suitable for Sfp	Reaction must be done in conditions suitable for BirA; requires ATP	Both tags must be at protein end (either N- and C- terminus); reaction must be done in conditions suitable for SnoopLigase	Large tag (SpyCatcher)	Noncovalent bond
Potential partners types	Protein–protein;	Protein–DNA;	Protein–surface;	Protein–protein;	Protein–protein;	Protein–surface;
	Protein-peptide;	Protein-peptide;	Protein purification (protein-resin);	Protein-peptide	Protein-peptide;	Protein-resin;
	Protein–DNA;	Protein-small compounds;	Protein–lipids;		Protein–surface	Protein-small compounds;
	Protein-fluorophore	Protein–surface	Protein–DNA			Protein-fluorophores;
						Protein–lipids
Examples of applications	Native antibody labeling;^217^	Tobacco mosaic virus decoration;^220^	Protein immobilization on the surface;^222,223^	Modular vaccine assembly;^226^	Formation of Nanoparticle Vaccine;^228^	Biosensors;^229^
	Modified antibody labeling;^218^	Protein–DNA conjugation^221^ for create large complexes or use for optical tweezers	Nanobody purification;^224^	Titin misfolding^227^	Titin misfolding^227^	Labeling proteins for MicroScale Thermophoresis (MST)^230^
	Encapsulin decoration^219^		Membrane protein activity studies^225^			

Another method is known as SpyTag/SpyCatcher (Table 6).^193,194^ This approach is notable as it allows for the spontaneous formation of an isopepetide bond between the SpyTag present on partner A and the SpyCatcher present on partner B. This system enables robust and site-specific protein conjugation without the need for additional enzymes or reagents, making it particularly useful for modular protein assembly.

Orthogonal methods aim to incorporate noncanonical (also known as unnatural) amino acids into the protein sequence during translation.^195^ This is done by supplementing the expression system with a specific amino acid, a matching aminoacyl-tRNA synthetase (aaRS), and a cognate tRNA. Libraries of hundreds of unnatural amino acids exists,^196–198^ offering diverse functional groups that enable various applications, including testing protein–protein interactions *in vitro* and *in vivo*,^199^ fluorescent labeling of protein in live neurons,^200^ investigation of protein post-translational modifications^201^ and protein fluorination for NMR studies.^202^ There are many excellent reviews^196–198,203–205^ for more in-depth coverage of this topic.

In contrast to covalent linkage strategies, noncovalent interactions are also commonly employed. These are typically based on high-affinity binding, such as the interaction between nickel ions and His-tags (see Table 6), which can provide reversible and tunable association between protein modules. In addition to these thoroughly characterized linkage strategies, it is becoming increasingly possible, using the protein and NA design tools described above, for proteins to be designed which bind small molecules, sugars, nucleic acids and other proteins in a bespoke way for the assembly of protein nanostructures.

Additionally, the design of membrane proteins is becoming increasingly feasible and as this technology develops, custom membrane proteins could be used to anchor to a target membrane.^206^ Moreover, recent cell-based engineered transmembrane receptor platforms demonstrate that membrane proteins can provide programmable sensing and signal transduction; analogous modules could, in principle, be adapted as functional membrane interfaces in bottom-up synthetic systems.^207,208^

## 4 Challenges and Future Prospects

We are at the beginning of a revolution in our ability to design and build programmable biological matter. On the design side, advances in software and hardware will likely lead to ever more convenient tools able to be utilized on cheaper computers. A widening of software capabilities is also desirable; design and prediction tools, historically used for peptide and protein analysis, and are being expanded rapidly to include nucleic acids and other nonproteinaceous atoms and molecules.^184,209^ It is likely that designing machine-like biological nanostructures with capabilities equally or exceeding those found in nature will require consideration of yet other classes of molecules such as lipids and here also progress is just beginning to be made.^210^ The ability to design and predict motion is a major challenge, the solving of which would be a further revolution in the field, for instance for synthetic cells.^211^

The ability to design artificial biological molecules (Figure 7) is already leading to real world outcomes notable in the medical field where the Skycovione vaccine used against SARS-CoV-2 for example is based on an artificial protein cage.^212,213^ Apart from vaccines, applications across the multiple medical fields including antibacterials, drug delivery systems, immune modulation *etc*. can all be envisaged and come with their own challenges to be solved such as the possibility of undesirable immune stimulation, unwanted clearance of designed nanoparticles *etc*. However, none of these challenges seem unsurmountable.

**Figure 7:**
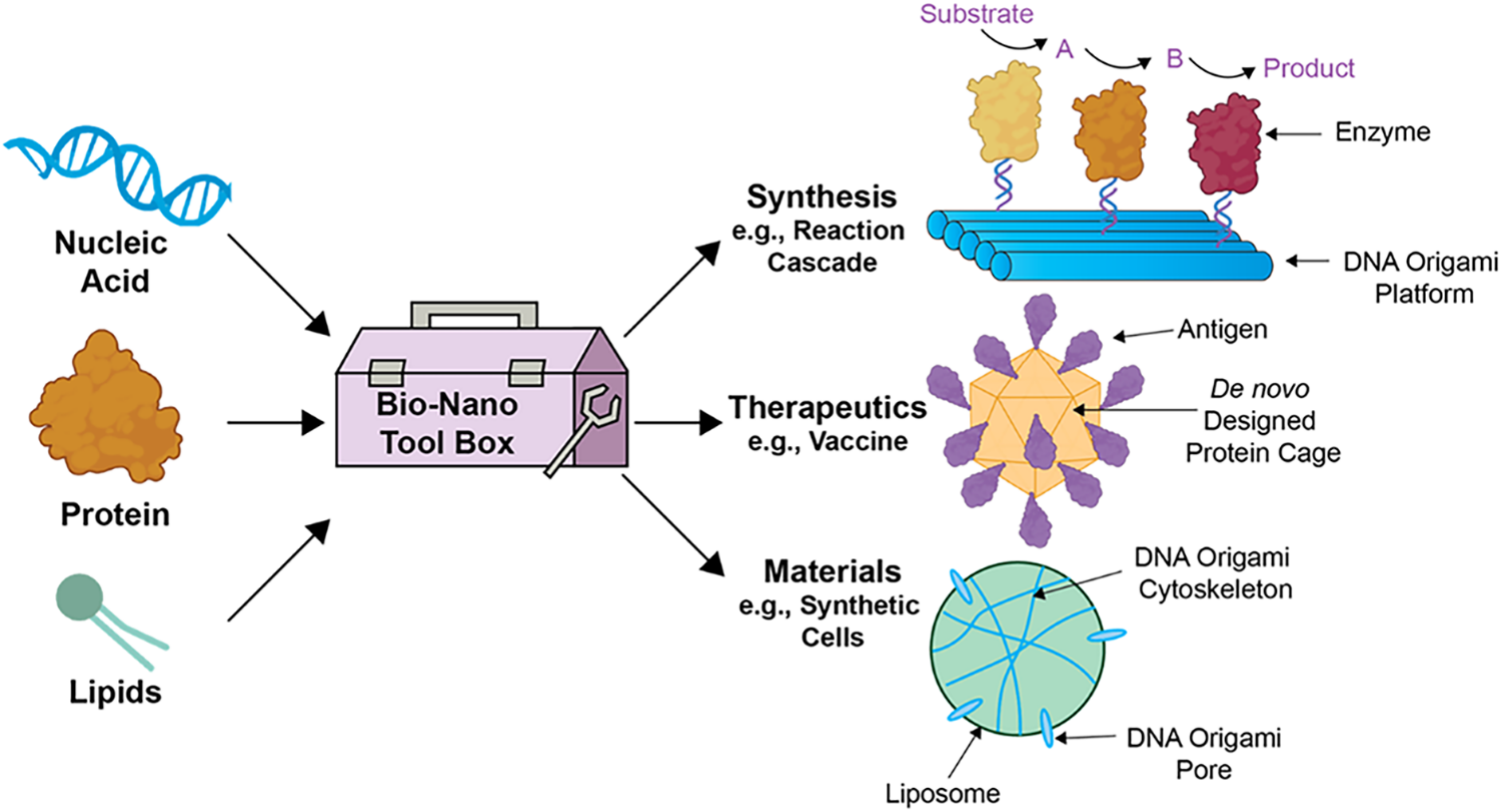
Examples of applications and uses of DNA nanotechnology, AI designed proteins and combinations with lipids. Reaction cascades for chemical synthesis can be set up with a DNA origami scaffold and enzymes.^215^ Therapeutic applications include drug delivery and vaccines, for example displaying viral antigens on the surface of a de novo designed protein cage.^212^ Novel materials such as bottom—up synthetic cells can be created by combining lipids with designed molecules, like DNA nanostructure pores and a DNA origami cytoskeleton.^214^

We expect to see increasing crossover between the fields of de novo nucleic acid and protein design. For example, while RNA origami structures are often produced and function *in situ* within cells this is not a common approach for de novo protein designs produced using the tools we have outlined. However, in light of technologies such as CRISPR-Cas that are produced and are active in target cells, there is potential that novel de novo designed proteins may be increasingly employed in a similar way.

Interoperability between different approaches to improve functional sophistication is likely to be increased. For example, the work mentioned above where DNA nanostructures were coated with protein structures could be adapted such the protein structures themselves were de novo designed, imparting new functionality. New AI-based tools for designing DNA-binding proteins are beginning to be developed.^209^

While medical use is an obvious and attractive avenue given the biological nature of the building materials, there may be yet unrealized major potential in other fields, for example, materials science. The vision of artificial cells is closer than ever with key components such as the membrane, membrane channels and pores and artificial cytoskeletons having been created with DNA nanostructures that interface with lipids (as discussed in other reviews^214^). Combining approaches, it may ultimately be possible to produce highly complex multicomponent systems that are able to demonstrate some of the desirable features of living cells.

Another exciting avenue that these tools have been used for is synthesis, where they can produce complex organic molecules more efficiently and with fewer harsh chemicals involved, by pushing enzyme catalysis to the next level. For example, by creating reaction cascades on a DNA scaffold to enhance the rate of catalysis.^215^ Tackling this challenge could play an important role in reducing the use of harsh chemicals and high temperatures required from some chemical synthesis routes. While safety and regulatory concerns must also be clearly considered given the potential power of these techniques and technologies, there are great prospects for major societal good as a result of the continued advancement of the field.

## Vocabulary

Wireframe DNA origami: Defines only the edges of a 3D shape unlike traditional rasterized DNA origami where faces are “filled in.”
DNA scaffold: In DNA origami, the long continuous strand that is molded into the required shape by numerous shorter staple strands.
Derjaguin-Muller-Toporov (DMT) modulus: A parameter derived from a simple adhesive contact mechanics model in atomic force microscopy (AFM) to estimate the elastic modulus of materials from tip–sample force data. Analyses the elastic contact with adhesion between the sample and the probe.
Generative diffusion: A method for generating viable data by denoising random noise. Commonly used in protein design to generate novel, protein structures from random noise.
Deep learning: A type of machine learning that uses multilayered artificial neural networks inspired by the human brain for data processing and identification of complex patterns. Used for the classification, prediction, and generation of data, or in this case, biological nanostructures.
